# Exploring the Anticancer Properties of 1,2,3-Triazole-Substituted Andrographolide Derivatives

**DOI:** 10.3390/ph18050750

**Published:** 2025-05-19

**Authors:** Joana R. L. Ribeiro, Juliana Calheiros, Rita A. M. Silva, Bruno M. F. Gonçalves, Carlos A. M. Afonso, Lucília Saraiva, Maria-José U. Ferreira

**Affiliations:** 1 Research Institute for Medicines (iMed.ULisboa), Faculty of Pharmacy, Universidade de Lisboa, Av. Prof. Gama Pinto, 1649-003 Lisbon, Portugal; 2 LAQV/REQUIMTE-Associated Laboratory for Green Chemistry (LAQV) of the Network of Chemistry and Technology (REQUIMTE), Laboratory of Microbiology, Department of Biological Sciences, Faculty of Pharmacy, Universidade do Porto, 4050-313 Porto, Portugal

**Keywords:** andrographolide, 1,2,3-triazole-substituted derivatives, antiproliferative, apoptosis, cell cycle arrest, antimigratory

## Abstract

**Background/Objectives**: The search for new anticancer agents from natural sources remains a key strategy in drug discovery. This study aimed to synthesize and evaluate novel triazole derivatives of the diterpenic lactone andrographolide for their antiproliferative activity against various cancer cell lines. **Methods**: Twenty-two new triazole derivatives (**5**–**26**), of the triacetyl derivative (**2**) of the diterpenic lactone andrographolide (**1**), were synthesized via the azide-alkyne “click reaction”. The antiproliferative effects of compounds **1**–**26** were evaluated using the sulforhodamine B assay against a panel of cancer cell lines and a non-tumorigenic colon cell line. A representative compound, triazole derivative **12**, was further evaluated in human pancreatic ductal adenocarcinoma (PANC-1) cells for its effects on the cell cycle, apoptosis, migration, and drug synergy with 5-fluorouracil. **Results**: Several compounds, specifically, **9**, **14**, **16,** and **17**, bearing a phenyl moiety, exhibited improved antiproliferative activity compared to the parental compound **1**. Derivative **12**, selected for further investigation, induced G2/M cell cycle arrest and apoptosis in a concentration-dependent manner. Additionally, this compound significantly reduced cell migration and demonstrated synergistic effects with 5-fluorouracil in PANC-1 cells. **Conclusions**: The synthesized andrographolide-based triazole derivatives, particularly compound **12**, showed promising antiproliferative activity and mechanisms relevant to cancer therapy. These findings support their potential as lead compounds for further development in anticancer research.

## 1. Introduction

Cancer remains one of the leading causes of mortality worldwide, accounting for approximately 10 million deaths in 2022 [1]. In addition, an increase in the number of cancer cases and deaths is expected due to factors such as population growth, aging, and the adoption of lifestyles that expose people to more cancer risk factors [2]. Despite significant and promising advancements in cancer treatment, conventional therapies still exhibit severe adverse effects and are frequently associated with resistance and relapse. These challenges are related with the highly heterogeneous nature of cancer as a disease, which consists of various cell types with different molecular characteristics and varied responses to treatment [3,4]. Consequently, there is an urgent need to explore alternative therapeutic approaches.

**Scheme 1:**
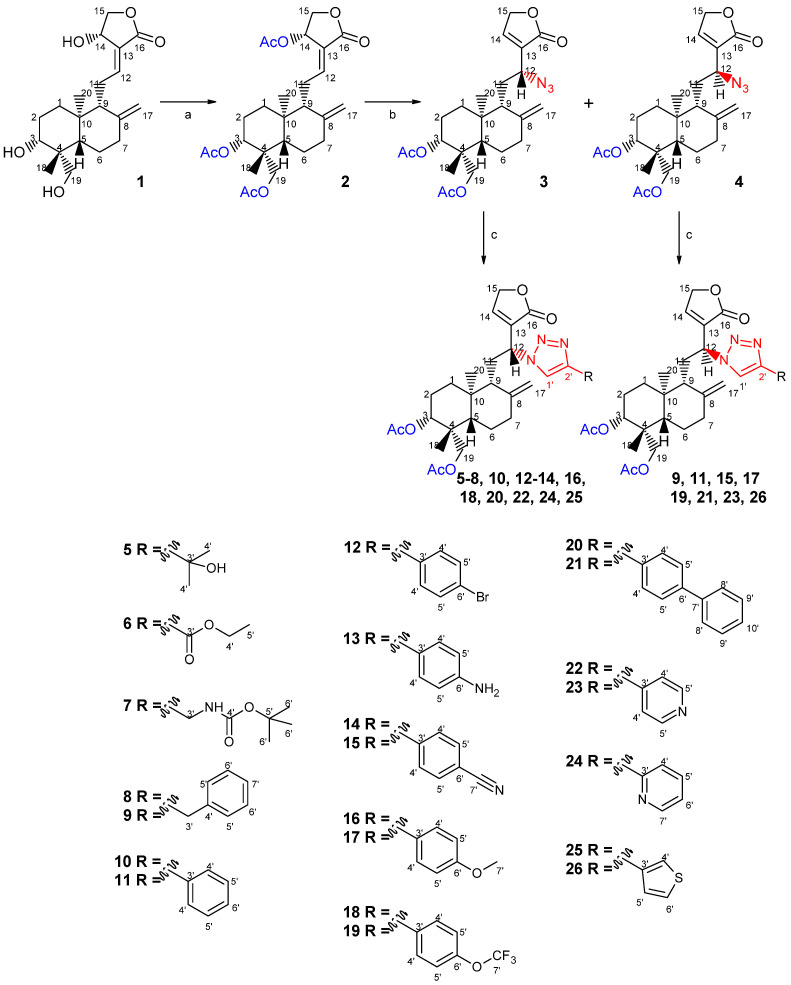
Preparation of triacetyl andrographolide (**2**), azide andrographolide (**3** and **4**), and triazole derivatives (**5**–**26**). Reagents and conditions: (a) acetic anhydride (40 equiv.), zinc chloride (0.13 equiv.), room temperature, 2 h, quantitative; (b) sodium azide (2.4 equiv.), methanol, room temperature, 1 day, **3**: 47%, **4**: 14%; (c) corresponding alkyne (1.2 equiv.), CuSO_4_.5H_2_O (5 mol%), sodium ascorbate (10 mol%), ethanol:water 50%, 50°C, 2 h–3 days, **5**: 38%, **6**: 31%, **7**: 52%, **8**: 46%, **9**: 49%, **10**: 37%, **11**: 31%, **12**: 83%, **13**: 8%, **14**: 47%, **15**: 36%, **16**: 43%, **17**: 18%, **18**: 51%, **19**: 25%, **20**: 76%, **21**: 44%, **22**: 44%, **23**: 21%, **24**: 88%, **25**: 82%, **26**: 21%.

Natural products have long been recognized for their potential in cancer management. Indeed, over 60% of currently used anticancer agents are derived from natural sources or inspired by natural compounds [5]. In this regard, one particularly promising natural compound is andrographolide (**1**, Scheme 1), a diterpene lactone found in the medicinal plant *Andrographis paniculate*, which has been used in traditional Chinese medicine [6]. The potential of andrographolide for the development of new antineoplastic drugs is clearly supported by the increasing number of publications reporting its anticancer effects over the last 15 years [7]. Among several other biological activities, numerous studies have demonstrated the ability of andrographolide to inhibit the growth and proliferation of various cancer cell lines, including those of breast [8], lung [9], prostate [10], and hematological origins [11], among others, as well as its antitumor properties in in vivo models. The anticancer properties of andrographolide have been attributed to several key factors: first, it exhibits the ability to modulate various signaling pathways and cellular processes involved in cancer progression, including NF-κB [12], STAT3 [13], and PI3K/Akt/mTOR [14]. This multitargeted nature allows it to interfere with various aspects of tumor development, survival, and proliferation. It is important to note that in the context of complex diseases, such as cancer, therapeutic approaches based on multifunctional drugs have shown improved efficacy over single-target agents [15,16]. While the underlying mechanisms of action of andrographolide have not yet been fully elucidated, ongoing research has identified some relevant anticancer mechanisms of andrographolide, which include induction of cell cycle arrest [17], promotion of apoptosis [17], inhibition of cancer cell proliferation [18], angiogenesis [19] and metastasis. Second, andrographolide has shown the ability to act synergistically with conventional chemotherapeutic agents [20], revealing its potential benefit as part of combination treatment plans. Third, it has demonstrated the capacity to overcome multidrug resistance in certain cancer cell lines [21,22]. Finally, the relative affordability and natural abundance of andrographolide makes this compound an ideal candidate for designing hit/lead compounds to develop novel anticancer agents.

Despite its enormous potential, andrographolide has been found to possess some limitations, namely its poor bioavailability, short half-life, and nonspecific cellular distribution [23,24], as well as some safety concerns that have been raised regarding nephrotoxicity [25] and reproductive toxicity [26].

Chemical modification of natural product scaffolds, including andrographolide, has proven to be a successful strategy to address these types of shortcomings by generating derivatives with improved potency, selectivity, and pharmacokinetic properties to become clinically useful drugs, particularly in the field of cancer treatment [27,28,29,30]. Previous structure–activity relationship (SAR) studies have shown that targeted modifications to specific positions on the andrographolide backbone can enhance both its biological activity and physicochemical properties, including solubility and bioavailability. These structural modifications have primarily focused on key functional sites such as the five-membered lactone ring (especially at positions C-14 and C-15), the C-12 position, and the hydroxyl groups at C-3 and C-19. Additional efforts have targeted the exocyclic double bond. SAR studies have consistently demonstrated that such modifications can significantly impact its cytotoxic activity [31]. For example, esterification of hydroxyl groups at C-3, C-14, C-17, and C-19 have been associated with improved anticancer activity relative to the parent compound [32,33,34,35]. Additionally, C-12-substituted aryl amino 14-deoxy-andrographolide derivatives have demonstrated enhanced cytotoxic effects across a range of cancer cell lines [36,37], underscoring the potential of this position for further optimization. More recently, the incorporation of an aryl-carbamate moiety at the C-14 position has yielded derivatives with potent anti-pancreatic cancer activity, attributed to downregulation of oncogenic p53 expression and inhibition of multiple malignant phenotypes in pancreatic cancer models [28]. While significant progress has been made, the andrographolide scaffold remains a versatile platform with further opportunities for structural diversification aimed at enhancing its therapeutic potential.

Click chemistry reactions, particularly the Cu(I)-catalyzed Huisgen 1,3-dipolar cycloaddition between alkynes and azides, were quickly recognized as a powerful tool for modifying various natural compounds. Indeed, this modification met the criteria established in 2001 by Professor K. Barry Sharpless, who coined the concept of “click chemistry” as the ideal strategy to modify natural products. These criteria include being reliable, selective, modular, wide in scope, giving very high yielding reactions, generating only inoffensive by-products that can be removed without chromatography, and being stereospecific [38,39]. This modification enables the direct incorporation of various functional groups accompanied by the formation of the multifunctional 1,2,3-triazole motif. Not only has this modification been shown to improve pharmacological activities, including anticancer properties, but it also enhances metabolic stability and drug-like properties [40,41,42]. These positive effects could be linked to the 1,2,3-triazole motif’s ability to act as a basic and hydrophilic connecting group or as bioisosteres of 5- or 6-membered heterocycles or an amide group. In addition, this motif can noncovalently interact with several biological targets through hydrogen bonds, dipole–dipole bonds, and van der Waals forces [43].

The introduction of the 1,2,3-triazole moiety into the andrographolide scaffold represents an opportunity to explore the generation and evaluation of novel andrographolide-1,2,3-triazole conjugates as potentially more effective anticancer candidates.

Thus, continuing our ongoing research program, which aims to find plant-derived compounds as anticancer agents for overcoming drug resistance [44,45,46,47,48], this work reports the generation of a library of new C-12 andrographolide-1,2,3-triazole hybrids. Herein, detailed information regarding the preparation protocol is provided, as well as the structural characterization data. All newly prepared derivatives underwent preliminary antiproliferative evaluation against a panel of cancer cell lines and a non-tumorigenic cell line. One of the compounds, compound **12**, was chosen for further experiments for a preliminary investigation of its mechanism of action.

## 2. Results and Discussion

### 2.1. Chemistry

Considering the reactivity of andrographolide (**1**) functional groups, a *γ*-butyrolactone moiety; a double bond between C-8 and C-17; another between C-12 and C-13; and three hydroxyl groups at C-3, C-14, and C-19, we envisioned the introduction of the azide moiety at C-12 as a starting point for the preparation of 1,2,3-triazole derivatives. Our approach, as outlined in Scheme 1, began with the protection of the hydroxyl groups. Andrographolide reacted with acetic anhydride in the presence of ZnCl_2_ to afford the intermediate triacetyl derivative **2** quantitatively. This transformation was selected as the first step to limit the high reactivity of hydroxyl groups and generate a better leaving group at C-14.

The C-12 position was then functionalized by reacting compound **2** with sodium azide in methanol (Scheme 1, reaction b). As expected, through Michael addition at C-12 followed by elimination of the acetyl group at C-14, a mixture of epimers was obtained, which was afterwards separated by column chromatography, yielding azide derivatives **3** and **4** in a ratio of 3:1 (yields of 47% and 14%, respectively). The structures of these derivatives were assigned based on their NMR data, including 2D-NMR experiments. When comparing the ^1^H and ^13^C-NMR spectra of compounds **3** and **4** with those of the triacetyl derivative **2**, as expected, the signal of the trisubstituted double bond H-12 (*δ*_H_ 6.99) and the corresponding carbon resonance (*δ*_C_ 150.1) were absent, whereas new signals of the geminal proton to the azide function and the corresponding carbon appeared at *δ*_H_ ≈ 4 and *δ*_C_ ≈ 56. Moreover, the new olefinic signal assigned to H-14, at *δ*_H_ 7.41 (**3**) and 7.37 (**4**), and the corresponding signal in the ^13^C-NMR spectrum, at *δ*_C_ 145.9 (**3**) and 147.4 (**4**), substantiated the formation of the new double bond at C-13, which was also corroborated by the absence of the proton and carbon signals of the acetyl group at C-14.

Further reaction of compounds **3** and **4** with different alkynes, by “click” chemistry [49], allowed the formation of twenty-two new 1,2,3-triazole derivatives (**5**–**26**) (Scheme 1, reaction c) in reduced-to-good yields (8–88%). The formation of the triazole ring was easily identified by the ^1^H-NMR data due to the presence of a singlet at *δ*_H_ 7.26 to 8.23. Additional proton and carbon signals, corresponding to the substituent of the triazole moiety, were also observed. Moreover, the signal corresponding to H-12, which was overlapped in both epimeric azines **3** and **4**, appeared in triazole derivatives as a well-defined doublet of doublets (*dd*, *J* ≅ 11.2, 3.5 Hz) (compounds **9**, **11**, **15**, **17 19**, **21**, **23**, **26**) or a broad doublet (*br d*, *J*_12-11_ ≅ 11.5 Hz) (compounds **5**–**8**, **10**, **12**–**14**, **18**, **20**, **22**, **24**, **25**), due to different *J*_12-11_ coupling patterns in both sets of epimers. The distinct *J*_12-11_ coupling patterns of both sets of derivatives is in agreement with other data from the literature [50], as well with our previous results of a library of nitrogen-containing andrographolide derivatives [22], where a well-defined doublet of doublets was associated with 12*S* configuration and a broad doublet to 12*R*. The stereochemistry at C-12 was also substantiated by nuclear Overhauser effects observed in NOESY experiments. Thus, in azide derivative **3**, a NOE correlation between H-12 and the H-9β, which was absent in the corresponding epimer **4**, agreed with a 12*R* configuration. Similarly, a strong NOE interaction between H-12/H-9 was also observed in the NOESY spectrum of compound **10**, which was selected as a representative of azide **3** derivatives, thus confirming the 12*R* configuration in this set of compounds. Conversely, as in azide derivative **4**, no nuclear Overhauser effect was found between H-12/H-9 in epimer **11**, in agreement with 12*S* stereochemistry for the set of azide **4** derivatives (see Supplementary Information, Figure S10).

### 2.2. Biological Studies

#### 2.2.1. In Vitro Antiproliferative Activity

The antiproliferative properties of andrographolide and all prepared derivatives (**2**–**26**) were evaluated against a panel of different cancer cell lines, including human pancreatic ductal adenocarcinoma (PANC-1), colorectal adenocarcinoma (HCT116), melanoma (A375), breast adenocarcinoma (MCF-7) and a non-tumorigenic colon (CCD-18Co) cell line using the sulforhodamine B assay. The cell lines were treated with increasing concentrations of each compound, and the IC_50_ (concentration that inhibits 50% of cell growth) values were determined after 48 h of incubation, as summarized in Table 1.

In general, most of the compounds showed significant antiproliferative activity, with IC_50_ values lower than 10 µM, in all the cancer cell lines. It was observed that the esterification of andrographolide hydroxyl groups with a lipophilic substituent significantly enhanced the antiproliferative activity in all cell lines tested, likely due to improved cellular permeability. When comparing with the parental compound (**1**, IC_50_ = 3.1 µM), the introduction of the triazole moiety increased the antiproliferative activity of compounds **9**, **14**, **16**, **17**, **20**, and **24** (IC_50_ ranging from 1.8 µM to 3.0 µM) against the PANC-1 cell line. Against the HCT116, A375, and MCF-7 cancer cell lines, most of the derivatives were found to have higher antiproliferative activity than the parental compound **1**. Except for compounds **3**, **5**, and **24**, all derivatives showed better antiproliferative activity, with IC_50_ ranging from 1.2 µM to 4.8 µM, against HCT116 cells than the parental compound **1** (IC_50_ = 5.1 µM). In A375 cells, the parental compound **1** (IC_50_ = 4.0 µM) also exhibited lower antiproliferative activity than most of the derivatives (IC_50_ ranging from 0.9 µM to 3.6 µM), with the exception of compounds **5**, **8**, **10**, **11**, **13**, **18**, and **19**. Again, in MCF-7 cells, only compounds **5**, **7**, **8**, **13**, and **20**, showed lower antiproliferative activity compared to the parental compound **1** (IC_50_ = 13.5 µM), while all the other compounds showed IC_50_ values ranging from 3.8 µM and 11.0 µM. Interestingly, the IC_50_ values for the compounds in MCF-7 cells were slightly higher compared to the other cancer cell lines tested. One possible explanation for the relatively higher IC_50_ values in MCF-7 cells may relate to intrinsic differences in cell line biology. MCF-7 is an estrogen receptor-positive (ER^+^) breast cancer cell line known to have a slower proliferation rate and a distinct apoptotic response compared to more aggressive and p53-deficient cell lines like PANC-1. Moreover, MCF-7 cells are deficient in caspase-3, a key executioner of apoptosis, which may partially attenuate the apoptotic response to the compounds and contribute to the reduced sensitivity.

In addition, the antiproliferative effect of the compounds was evaluated on the non-tumorigenic (CCD-18Co) cell line. Comparison of the IC_50_ values in the CCD-18Co cells with those obtained in cancer cell lines revealed that most compounds exhibited some degree of selectivity towards the PANC-1, HCT116, and A375 cell lines. In contrast, only a few compounds exhibited modest selectivity in the MCF-7 cell line.

Compound **12** was selected as a representative compound for further studies in the pancreatic ductal adenocarcinoma (PANC-1) cells to carry out preliminary exploration of its mechanism of action. This compound was selected based on two main factors: its representative biological profile and its availability in greater quantity. This cell line was selected considering that pancreatic ductal adenocarcinoma is a lethal solid malignancy with limited therapeutic options.

#### 2.2.2. Cell Cycle Assay

**Figure 1:**
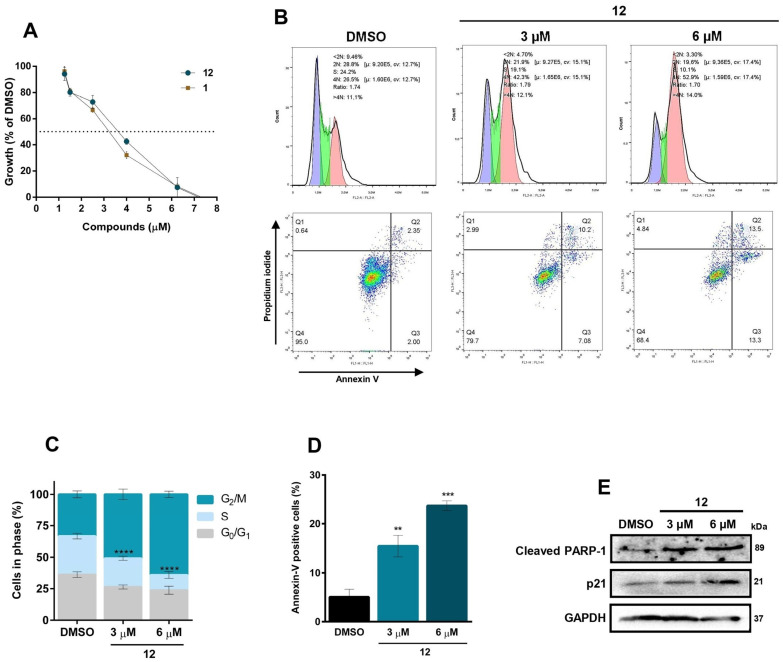
Growth inhibitory effect of compound **12** on cancer cell growth, associated with cell cycle arrest and apoptosis, in PANC-1 cells. (**A**) Dose–response curve for the growth inhibitory effect of compounds **1** and **12** on PANC-1 cells, after 48 h of treatment; growth obtained with control (DMSO) was set as 100%; data are mean ± SEM of three independent experiments (two replicates each). The dotted line represents 50% of growth inhibition. (**B**) Representative flow cytometry histograms of the percentage of PANC-1 cells treated for 48 h with DMSO, 3 and 6 µM of compound 12. In the upper panel, representative flow cytometry histograms of cell cycle (G0/G1 phases in purple, S phase in green, and G2/M phases population in orange); in the bottom panel, representative flow cytometry histograms of apoptosis (viable cells: annexin-V−, PI−, lower left quadrant; early apoptosis: annexin-V+, PI−, lower right quadrant; late apoptosis: annexin-V+, PI+, upper right quadrant; necrosis: annexin-V−, PI+, upper left quadrant). (**C**) Effect of 3 and 6 µM of compound **12** on cell cycle progression in PANC-1 cells, after 48 h of treatment; the percentage of cells in each phase of the cell cycle was analyzed by flow cytometry, using propidium iodide (PI); data are mean ± SEM of three independent experiments; **** *p* < 0.0001, significantly different from DMSO (two-way ANOVA with Dunnett’s test). (**D**) Effect of 3 and 6 µM of compound **12** on apoptosis in PANC-1 cells, after 48 h of treatment; the percentage of annexin-positive cells was analyzed by flow cytometry using PI and annexin-V staining; data are mean ± SEM of three independent experiments; ** *p* < 0.01 and *** *p* < 0.001, significantly different from DMSO (one-way ANOVA with Dunnett’s test). (**E**) Effect of 3 and 6 µM of compound **12** on expression levels of cleaved PARP-1 and p21 after 48 h of treatment, in PANC-1 cells. Representative immunoblots are shown. GAPDH was used as a loading control.

To elucidate the antiproliferative activity of compound **12**, cell cycle analysis was performed in the PANC-1 cancer cell line. The cell cycle is a highly controlled process where cellular DNA is replicated and then equally partitioned in two daughter cells. The cell cycle is composed of two distinct phases: the interphase, where the cell grows and undergoes three distinct periods—the G1 (pre-DNA synthesis), S (DNA synthesis), and G2 (pre-division) phases—and mitosis, where cell undergoes division. The newly formed daughter cells then enter the preparatory phases of interphase, and start the G0 phase (quiescence) [51]. As shown in Table 1 and Figure 1A, compound **12** exhibited an IC_50_ value of 3.4 ± 0.9 µM, after 48 h of treatment. Thus, PANC-1 cells were treated with compound **12** at 3 and 6 µM for 48 h and then analyzed by flow cytometry (Figure 1B,C). Treatment with 3 μM and 6 μM resulted in a significant increase in the G2/M population compared to the control (Figure 1B, upper panel; and Figure 1C), indicating that this compound can induce cell cycle arrest at this phase, which is consistent with its observed cell growth inhibition properties. Considering these results, we explored the effects of this compound on the level of the cell cycle-regulatory protein, the cyclin-dependent kinase (CDK) inhibitor p21, by Western blot analysis. The results showed that treatment of PANC-1 cells with 3 and 6 µM of compound **12** for 48 h increased the expression of p21 in a dose-dependent manner (Figure 1E). Considering that p21 inhibits the kinase activity of CDK2/cyclin-E complexes, which promote the progression of the G2 phase of the cell cycle, our findings suggest that the upregulation of p21 induced by compound **12** led to cell cycle arrest at the G2/M phase, resulting in the inhibition of cell proliferation.

#### 2.2.3. Annexin V-FITC/PI Double-Staining Assay

To further investigate the mechanism of action of the triazole derivative **12**, an apoptosis analysis was subsequently performed in PANC-1 cells. The early stages of apoptosis are characterized by the disruption of membrane asymmetry and the translocation of phosphatidylserine from the inner to the outer leaflet of the plasma membrane, which can be detected using Annexin V-FITC, a protein with high affinity for phosphatidylserine. In the later stages of apoptosis, the cell membrane loses its integrity, allowing propidium iodide (PI) to penetrate into the cell [52]. Thus, PANC-1 cells treated with compound **12** at 3 and 6 μM for 48 h were analyzed by flow cytometry after Annexin V-FITC and PI staining (Figure 1B, bottom panel; and Figure 1D). This analysis can distinguish and quantify viable cells (annexin V−, PI−), early apoptotic cells (annexin V+, PI−), late apoptotic cells (annexin V+, PI+), and necrotic cells (annexin V−/PI+). As observed in Figure 1B, treatment with compound **12** led to a significant increase in the percentage of apoptotic cells (Annexin V+) from 4.35% (2% early apoptotic and 2.35% late apoptotic) in DMSO-treated cells to 17.28% (7.08% early apoptotic and 10.2% late apoptotic) and 26.8% (13.3% early apoptotic and 13.5% late apoptotic) at 3 and 6 µM, respectively. These results suggest that compound **12** exhibited potential as an apoptotic inducer, which led us to evaluate the effect of compound **12** on the levels of cleaved poly(ADP-ribose)polymerase 1 (PARP-1). Cleavage of PARP-1 into an 89 kDa protein, cleaved PARP-1, occurs during programmed cell death (apoptosis) as a result of proteolytic processing by active caspase-3. Thus, cleaved PARP-1 is recognized as a marker of apoptosis [53]. As shown in Figure 1E, treatment with 3 and 6 μM of compound **12** induced a significant increase in the levels of cleaved PARP-1, confirming its ability to induce apoptosis.

#### 2.2.4. Scratch (Wound Healing) Assay

**Figure 2:**
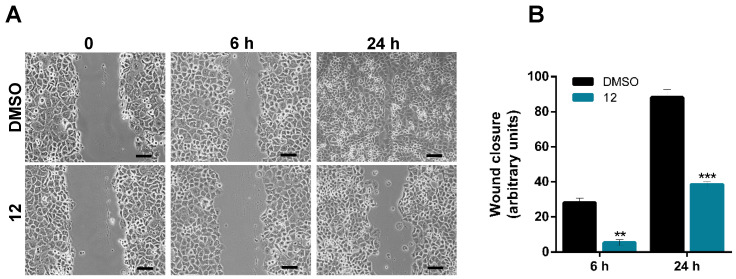
Effect of compound **12** on cancer cell migration. Effect of 1.35 µM of compound **12** (or DMSO only) on confluent PANC-1 cell migration after 6 and 24 h of treatment. In (**A**), representative figures are shown; scale bar = 100 µm, magnification 100×. In (**B**), quantification of wound closure using randomly selected microscopic fields (six fields per sample) is shown. Data are mean ± SEM of four independent experiments, values significantly different from DMSO: ** *p* < 0.01, *** *p* < 0.001 (two-way ANOVA with Sidak’s test).

Metastasis remains the greatest challenge in the clinical management of cancer. Cell migration is a key process that, during the whole cascade of cancer development, contributes to tumor invasion and metastasis. The scratch assay is a common in vitro technique used to assess two-dimensional cell migration and motility. It involves creating an artificial scratch or “wound” in a confluent monolayer of cells and then monitoring the progression of the wound over time [54,55]. Therefore, a wound healing assay was used to evaluate the effect of compound **12** on the cell migration ability of PANC-1 cells. Treatment with 1.35 µM (IC_10_, a concentration which has no significant effect on cell proliferation) of compound **12** significantly reduced the wound closure in PANC-1 within a period of 6 h and 24 h compared to control (Figure 2A,B). These data suggest that compound **12** exhibits anti-migratory activity in PANC-1 cells.

#### 2.2.5. Drug Combination Assay

**Figure 3:**
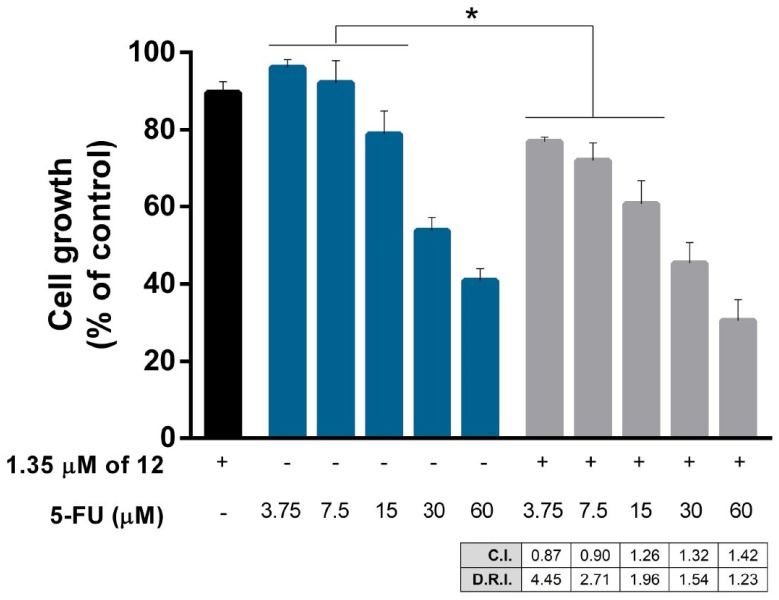
Synergistic effects of compound **12** with 5-FU, in PANC-1 cells. Effect of 1.35 µM of compound **12**, alone and in combination with a concentration range of 5-FU, in PANC-1 cell proliferation, determined by SRB assay after 48 h of treatment. Growth obtained with DMSO was set as 100%; data are mean ± SEM of six independent experiments; growth significantly different from 5-FU alone: * *p* < 0.05 (two-way ANOVA with Sidak’s test). CI and DRI values were obtained using CompuSyn software (CI < 1, synergy; 1 < CI < 1.1, additive effect; CI > 1.1, antagonism).

For the combination studies, 5-fluorouracil (5-FU) was chosen based on its clinical relevance and frequent use as a chemotherapeutic agent in the treatment of several cancers represented in our study, particularly colorectal (HCT116) and breast cancer (MCF-7). While 5-FU is not the first-line agent for pancreatic cancer, it has been used in certain treatment regimens in combination therapies such as FOLFIRINOX. However, resistance to 5-FU through diverse mechanisms is commonly reported, presenting a challenge to effective treatment. One of the approaches that have been explored to overcome this resistance and enhance the therapeutic efficacy is to combine 5-FU with other chemotherapeutic agents and/or alternative treatment approaches. Therefore, the potential synergistic effect of compound **12** in combination with 5-FU was evaluated by treating PANC-1 cancer cells with 1.35 µM of compound **12**, alone and in combination with a concentration range of 5-FU (3.75–60 µM) for 48 h. Using the CompuSyn software (version 1.0), a multiple drug effect analysis was performed for each combination, to determine combination index (CI) and dose-reduction index (DRI) values. CI values indicate the effect of combining multiple drugs as synergistic (CI < 1), additive (CI = 1), or antagonistic (CI > 1). DRI values represent the fold decrease in the dose of a drug needed when in combination to achieve the same efficacy as the drug alone. DRI values > 1 are favorable given the concern for toxicity when combining multiple drugs. As observed in Figure 3, a synergistic effect was observed between compound **12** and 5-FU at 3.75 and 7.5 µM, with CI values of 0.87 and 0.90, respectively. The DRI values validated this effect, since the higher values were obtained for the combinations of compound **12** with 3.75 and 7.5 µM of 5-FU. These findings indicated that the combination of compound **12** with 5-FU could be a promising strategy to improve the therapeutic efficacy of 5-FU, re-sensitizing pancreatic cancer cells to its effect. Importantly, at higher concentrations of 5-FU, the CI values shift toward additivity or mild antagonism. This phenomenon is not uncommon in combination therapy studies and may reflect dose-dependent pharmacodynamic interactions. At lower concentrations, compound **12** may potentiate the effects of 5-FU through complementary mechanisms, such as enhancing cell cycle arrest or increasing sensitivity to DNA damage. However, at higher concentrations, factors such as saturation of cytotoxic pathways, activation of stress responses, or overlapping toxicities may diminish the combinatorial advantage, resulting in reduced efficacy or antagonistic effects. Collectively, these findings highlight that the most pronounced synergistic effects occur at lower doses, which could be advantageous for developing combination regimens that maintain efficacy while minimizing toxicity.

## 3. Materials and Methods

### 3.1. Chemistry

#### 3.1.1. General Remarks

Andrographolide was purchased from Sigma Aldrich (Steinheim, Germany), in over 90% purity. Reagent-grade chemicals and analytical-grade solvents used in reactions and workups were purchased from Alfa Aesar (Heysham, Lancashire, UK), Sigma-Aldrich (Steinheim, Germany), or Merck (Darmstadt, Germany). Deuterated chloroform used for NMR analysis was purchased from Merck (Darmstadt, Germany) with a degree of purity higher than 95%. Column chromatography was performed using silica gel 230–400 mesh ATM (Merck, (Darmstadt, Germany). Thin-layer chromatography (TLC), used to monitor all reactions, was carried out on pre-coated silica gel 60 F254 (Merck, Darmstadt, Germany) and visualized under UV light (λ 254 and 366 nm) and/or by exposure to a solution of sulfuric acid–methanol (1:1) followed by heating. Preparative thin-layer chromatography was performed with silica gel 60 GF254 0.040–0.063 mm (Merck, Darmstadt, Germany). The 1D and 2D NMR spectra were recorded on a Bruker Fourier 300 Ultra-Shield spectrometer (Bruker, Rheinstetten, Germany), using CDCl_3_ as internal standard. Chemical shifts (*δ*) are reported in parts per million (ppm), and coupling constants (*J*) in hertz (Hz). Multiplicities are given as s (singlet), d (duplet), t (triplet), q (quartet), quint (quintuplet), and m (multiplet). Spectra were assigned using appropriate COSY, DEPT, HSQC, and HMBC sequences. The low-resolution mass spectrometry was performed using a Waters AcquityTM triple quadruple spectrometer (Waters, Wilmslow, UK).

#### 3.1.2. NMR Data of Andrographolide (**1**)

White powder; ^1^H-NMR (300 MHz, CDCl_3_) δ = 6.62 (1H, *td*, *J* = 6.8, 1.7 Hz, H-12), 5.70 (1H, *d*, *J* = 6.1 Hz, H-14-OH), 5.04 (1H, *d*, *J* = 4.9 Hz, H-3-OH), 4.91 (1H, *br t*, *J* = 6.1 Hz, H-14), 4.81 (1H, *br s*, H-17a), 4.63 (1H, *br s*, H-17b), 4.39 (1H, *dd*, *J* = 9.9, 6.1 Hz, H-15a), 4.12 (1H, *dd*, *J* = 7.5, 2.9 Hz, H-19-OH), 4.03 (1H, *dd*, *J* = 9.9, 2.1 Hz, H-15b), 3.84 (1H, *dd*, *J* = 11.0, 2.9 Hz, H-19a), 3.27 (2H, *m*, H-3 and H-19b), 2.46 (2H, *m*, H-11), 2.32 (2H, *m*, H-7), 1.95 (2H, *m*, H-2), 1.74 (2H, *m*, H-6), 1.63 (2H, *m*, H-1), 1.30 (1H, *m*, H-9), 1.20 (1H, *m*, H-5), 1.08 (3H, *s*, H-18), 0.66 (3H, *s*, H-20) ppm. ^13^C-NMR (75 MHz, DMSO-d6) δ = 169.9 (C-16), 147.6 (C-12), 146.3 (C-8), 129.0 (C-13), 108.2 (C-17), 78.4 (C-3), 74.3 (C-15), 64.5 (C-14), 62.6 (C-19), 55.5 (C-9), 54.4 (C-5), 42.3 (C-4), 38.6 (C-10), 37.5 (C-7), 36.5 (C-1), 27.9 (C-2), 23.9 (C-6 and C-11), 23.0 (C-18), 14.7 (C-20) ppm. ESI-MS (positive mode) *m*/*z* (rel. Int) 477 [M + H]^+^. These data agree with the literature [56,57].

#### 3.1.3. Preparation of 3,14,19-Triacetyl Andrographolide (**2**)

Zinc chloride (76 mg, 0.56 mmol) was added to a solution of andrographolide (**1**, 1.5 g, 4.28 mmol) in acetic anhydride (16.2 mL, 171.2 mmol). The mixture was stirred for 2 h at room temperature. After completing the reaction, the excess acetic anhydride evaporated. The crude reaction was dissolved in dichloromethane:water = 1:1. The organic layer was then washed with a saturated solution of sodium bicarbonate, water, and brine; dried over anhydrous sodium sulphate; filtered; and evaporated. The compound was obtained as a white amorphous powder (quantitative yield). ^1^H-NMR (300 MHz, CDCl_3_) *δ* = 6.99 (1H, td, *J* = 6.8, 1.7 Hz, H-12), 5.91 (1H, dt, *J* = 6.2, 1.7 Hz, H-14), 4.89 (1H, br s, H-17a), 4.60 (1H, dd, *J* = 11.2, 4.3 Hz, H-3), 4.52 (1H, dd, *J* = 11.2, 6.2 Hz, H-15a), 4.51 (1H, br s, H-17b), 4.34 (1H, d, *J* = 11.8 Hz, H-19a), 4.23 (1H, dd, *J* = 11.2, 1.8 Hz, H-15b), 4.11 (1H, d, *J* = 11.8 Hz, H-19b), 2.10 (3H, s, H-14OCOCH_3_), 2.03 (6H, s, H-3OCOCH_3_ and H-19OCOCH_3_), 1.32 (1H, m, H-9), 1.02 (3H, s, H-18), 0.74 (3H, s, H-20) ppm. ^13^C-NMR (75 MHz, CDCl_3_) *δ* = 170.9 (C-19OCOCH_3_), 170.5 (C-3OCOCH_3_), 170.4 (C-14OCOCH_3_), 169.0 (C-16), 150.1 (C-12), 146.5 (C-8), 124.0 (C-13), 108.9 (C-17), 79.6 (C-3), 71.6 (C-15), 67.8 (C-14), 64.7 (C-19), 55.8 (C-5), 55.2 (C-9), 41.3 (C-4), 38.9 (C-10), 37.8 (C-7), 37.0 (C-1), 25.2 (C-11), 24.6 (C-6), 24.2 (C-2), 22.7 (C-18), 21.1 (C-3OCOCH_3_ and C-19OCOCH_3_), 20.7 (C-14OCOCH_3_), 14.5 (C-20) ppm. ESIMS *m*/*z* 477 [M + H]^+^.

#### 3.1.4. Preparation of the Azide Andrographolide Derivatives

Preparation of azide-containing derivatives (**3** and **4**) was as follows: compound **2** (300 mg, 0.63 mmol) was dissolved in methanol (3 mL) and then the sodium azide (123 mg, 1.89 mmol) was added. The reaction mixture was stirred for 1 day at room temperature. After evaporation of the solvent, the residue was purified by column chromatography (toluene:EtOAc, 10:0 to 5:5) to afford compounds **3** (135 mg, 47%) and **4** (41 mg, 14%) as a yellow amorphous powder.

*(12R)-azido-3,19-diacetoxy-14-deoxy-andrographolide* (**3**): ^1^H-NMR (300 MHz, CDCl3) δ = 7.41 (1H, *q*, *J* = 1.6 Hz, H-14), 5.00 (1H, *s*, H-17a), 4.90 (1H, *s*, H-17b), 4.88 (2H, *m*, H-15 a,b), 4.64 (1H, *dd*, *J* = 11.5, 4.7 Hz, H-3), 4.38* (1H, *m*, H-12), 4.38 (1H, *d*, *J* = 11.7 Hz, H-19a), 4.11 (1H, *d*, *J* = 11.7 Hz, H-19b), 2.05 (6H, *s*, H-3OCOCH_3_ and H-19OCOCH_3_), 1.54 (1H, *m*, H-9), 1.05 (3H, *s*, H-18), 0.76 (3H, *s*, H-20) ppm. ^13^C-NMR (75 MHz, CDCl_3_) δ = 172.1 (C-16), 171.0 (C-19OCOCH_3_), 170.6 (C-3OCOCH_3_), 145.9 (C-14), 145.8 (C-8), 134.2 (C-13), 108.6 (C-17), 79.8 (C-3), 70.5 (C-15), 64.7 (C-19), 56.8 (C-12), 55.2 (C-5), 52.3 (C-9), 41.2 (C-4), 38.9 (C-10), 38.2 (C-7), 36.7 (C-1), 28.8 (C-11), 24.8 (C-6), 24.2 (C-2), 22.6 (C-18), 21.2 (C-3OCOCH_3_), 21.1 (C-19OCOCH_3_), 14.7 (C-20) ppm. ESIMS *m*/*z* 460 [M + H]^+^. *Overlapped with H-19a.
*(12S)-azido-3,19-diacetoxy-14-deoxy-andrographolide* (**4**): ^1^H-NMR (300 MHz, CDCl_3_) δ = 7.37 (1H, *br s*, H-14), 4.92 (1H, *s*, H-17a), 4.89 (2H, *br s,* H-15 a,b), 4.63 (1H, *s*, H-17b), 4.55 (1H, *dd*, *J* = 11.3, 5.1 Hz, H3), 4.36* (1H, *dd*, *J* = 8,4, 3,8 Hz, H-12), 4.33 (1H, *d*, *J* = 11.7 Hz, H-19a), 4.08 (1H, *d*, *J* = 11.7 Hz, H-19b), 2.02 (6H, *s*, H-3OCOCH_3_ and H-19OCOCH_3_), 1.51 (1H, *m*, H-9), 0.98 (3H, *s*, H-18), 0.71 (3H, *s*, H-20) ppm. ^13^C-NMR (75 MHz, CDCl_3_) δ = 172.0 (C-16), 170.9 (C-19OCOCH_3_), 170.6 (C-3OCOCH_3_), 147.4 (C-14), 146.5 (C-8), 132.9 (C-13), 107.6 (C-17), 79.7 (C-3), 70.3 (C-15), 64.7 (C-19), 56.2 (C-12), 55.3 (C-5), 52.9 (C-9), 41.3 (C-4), 39.3 (C-10), 38.1 (C-7), 36.8 (C-1), 28.0 (C-11), 24.8 (C-6), 24.2 (C-2), 22.6 (C-18), 21.2 (C-3OCOCH_3_), 21.1 (C-19OCOCH_3_), 14.6 (C-20) ppm. ESIMS *m*/*z* 460[M + H]^+^. *Overlapped with H-19a.

#### 3.1.5. Preparation of Triazole-Containing Andrographolide Derivatives

Compounds **3** and **4** (0.044–0.78 mmol) were dissolved in EtOH: MeOH 5:5 and then the copper sulphate pentahydrate (5% mol), the sodium ascorbate (10 mol %), and the appropriate alkyne (1.2 equiv.) were added. The reaction mixture was stirred for 2 h–3 days at 50–60 °C. After evaporating the solvent, the residue was dissolved in acetonitrile, filtered, and purified by column chromatography (hexane:EtOAc, 1:0 to 1:1).

*(12R)-4-(2-methyl-2-ol)-1,2,3-triazole-3,19-diacetoxy-14-deoxy-andrographolide* (**5**): Obtained from reaction of compound **3** (30 mg, 0.065 mmol) with 2-methyl-3-butyn-2-ol (7.6 µL, 0.78 mmol). The residue was purified by column chromatography to afford compound **5** (14 mg, 38%) as a white amorphous powder. ^1^H-NMR (300 MHz, CDCl_3_) δ = 7.49 (1H, *s*, H-1′), 7.26 (1H, *q*, *J* = 1.5 Hz, H-14), 5.45 (1H, *br d*, *J* = 11.9 Hz, H-12), 5.03 (1H, *s*, H-17a), 4.94 (1H, *s*, H-17b), 4.80* (1H, *dt*, *J* = 18.5, 1.5 Hz, H-15a), 4.69* (1H, *dt*, *J* = 18.5, 1.7 Hz, H-15b), 4.48 (1H, *dd*, *J* = 11.5, 5.0 Hz, H-3), 4.31 (1H, *d*, *J* = 11.8 Hz, H-19a), 4.06 (1H, *d*, *J* = 11.8 Hz, H-19b), 2.01 (3H, *s*, H-3OCOCH_3_), 2.00 (3H, *s*, H-19OCOCH_3_), 1.65 (6H, *s*, H-4′), 1.11 (1H, *m*, H-9), 0.94 (3H, *s*, H-18), 0.73 (3H, *s*, H20) ppm. *AB part of an ABXY spin system with similar coupling values of A (*J* ≅ 1.5) and B (*J* ≅ 1.7) to X and Y. ^13^C-NMR (75 MHz, CDCl_3_) δ = 171.9 (C-16), 170.9 (C-19OCOCH_3_), 170.4 (C-3OCOCH_3_), 155.4 (C-2′), 147.9 (C-14), 145.8 (C-8), 133.6 (C-13), 120.2 (C-1′), 108.5 (C-17), 79.5 (C-3), 70.5 (C-15), 68.5 (C-3′), 64.7 (C-19), 55.7 (C-12), 55.1 (C-5), 51.9 (C-9), 41.2 (C-4), 38.9 (C-10), 38.2 (C-7), 36.4 (C-1), 30.4 (C-4′), 28.9 (C-11), 24.8 (C-6), 24.1 (C-2), 22.3 (C-18), 21.1 (C-3OCOCH_3_), 21.0 (C-19OCOCH_3_), 14.7 (C-20) ppm. ESIMS *m*/*z* 544 [M + H]^+^.
*(12R)-4-propriolate-1,2,3-triazole-3,19-diacetoxy-14-deoxy-andrographolide* (**6**): Obtained from reaction of compound **3** (30 mg, 0.065 mmol) with 2-ethynylpyridine (7.9 µL, 0.78 mmol). The residue was purified by column chromatography to afford compound **6** (11 mg, 31%) as a white amorphous powder. ^1^H-NMR (300 MHz, CDCl_3_) δ = 8.14 (1H, *s*, H-1′), 7.37 (1H, *q*, *J* = 1.5 Hz, H-14), 5.55 (1H, *br d*, *J* = 11.6 Hz, H-12), 5.04 (1H, *s*, H-17a), 4.91 (1H, *s*, H-17b), 4.92* (1H, *dt*, *J* = 18.6, 1.5 Hz, H-15a), 4.77* (1H, *dt*, *J* = 18.6, 1.7 Hz, H-15b), 4.47 (1H, *dd*, *J* = 11.7, 4.9 Hz, H-3), 4.44 (2H, *q*, *J* = 7.1 Hz, H-4′), 4.30 (1H, *d*, *J* = 11.8 Hz, H-19a), 4.06 (1H, *d*, *J* = 11.8 Hz, H-19b), 2.02 (3H, *s*, H-3OCOCH_3_), 2.00 (3H, *s*, H-19OCOCH_3_), 1.43 (3H, *t*, *J* = 7.1 Hz, H-5′), 1.14 (1H, *m*, H-9), 0.94 (3H, *s*, H-18), 0.73 (3H, *s*, H-20) ppm. *AB part of an ABXY spin system with similar coupling values of A (*J* ≅ 1.5) and B (*J* ≅ 1.7) to X and Y. ^13^C-NMR (75 MHz, CDCl_3_) δ = 171.7 (C-16), 170.9 (C-19OCOCH_3_), 170.3 (C-3OCOCH_3_), 160.5 (C-3′), 148.1 (C-14), 145.6 (C-8), 140.2 (C-2′), 132.9 (C-13), 128.2 (C-1′), 118.6 (C-7′), 108.6 (C-17), 79.3 (C-3), 70.6 (C-15), 64.7 (C-19), 61.6 (C-4′), 56.4 (C-12), 54.9 (C-5), 51.7 (C-9), 41.2 (C-4), 38.9 (C-10), 38.1 (C-7), 36.4 (C-1), 28.8 (C-11), 24.8 (C-6), 24.0 (C-2), 22.2 (C-18), 21.1 (C-3OCOCH_3_), 21.0 (C-19OCOCH_3_), 14.7 (C-20), 14.3 (C-5′) ppm. ESIMS *m*/*z* 558 [M + H]^+^.
*(12R)-4-(tert-butyl methyl carbamate)-1,2,3-triazole-3,19-diacetoxy-14-deoxy-andrographolide* (**7**): Obtained from reaction of compound **3** (30 mg, 0.065 mmol) with *N*-*boc*-propargylamine (12 mg, 0.78 mmol). The residue was purified by column chromatography to afford compound **7** (21 mg, 52%) as a white amorphous powder. ^1^H-NMR (300 MHz, CDCl_3_) δ = 7.53 (1H, *s*, H-1′), 7.28 (1H, *q*, *J* = 1.5 Hz, H-14), 5.45 (1H, *br d*, *J* = 11.5 Hz, H-12), 5.03 (1H, *s*, H-17a), 4.96 (1H, *s*, H-17b), 4.85* (1H, *dt*, *J* = 18.5, 1.5 Hz, H-15a), 4.76* (1H, *dt*, *J* = 18.5, 1.7 Hz, H-15b), 4.48 (1H, *dd*, *J* = 11.3, 4.9 Hz, H-3), 4.42 (2H, *d*, *J* = 6.0 Hz, H-3′), 4.30 (1H, *d*, *J* = 11.7 Hz, H-19a), 4.05 (1H, *d*, *J* = 11.7 Hz, H-19b), 2.01 (3H, *s*, H-3OCOCH_3_), 2.00 (3H, *s*, H-19OCOCH_3_), 1.43 (9H, *s*, H-6′), 1.11 (1H, *m*, H-9), 0.92 (3H, *s*, H-18), 0.75 (3H, *s*, H-20) ppm. *AB part of an ABXY spin system with similar coupling values of A (*J* ≅ 1.5) and B (*J* ≅ 1.7) to X and Y. ^13^C-NMR (75 MHz, CDCl_3_) δ = 171.8 (C-16), 170.9 (C-19OCOCH_3_), 170.6 (C-3OCOCH_3_), 147.7 (C-14), 145.7 (C-8), 145.3 (C-4′), 130.2 (C-2′), 122.8 (C-1′), 133.5 (C-13), 108.6 (C-17), 79.9 (C-5′), 79.4 (C-3), 70.5 (C-15), 64.7 (C-19), 55.8 (C-12), 55.0 (C-5), 51.8 (C-9), 41.2 (C-4), 38.9 (C-10), 38.2 (C-7), 36.4 (C-1), 36.1 (C-3′), 28.9 (C-11), 28.4 (C-6′), 24.8 (C-6), 24.1 (C-2), 22.3 (C-18), 21.1 (C-3OCOCH_3_), 21.0 (C-19OCOCH_3_), 14.7 (C-20) ppm. ESIMS *m*/*z* 615 [M + H]^+^.
*(12R)-4-benzyl-1,2,3-triazole-3,19-diacetoxy-14-deoxy-andrographolide* (**8**): Obtained from reaction of compound **3** (30 mg, 0.065 mmol) with 3-phenyl-1-propyne (9.7 µL, 0.78 mmol). The residue was purified by column chromatography to afford compound **8** (17 mg, 46%) as a white amorphous powder. ^1^H-NMR (300 MHz, CDCl_3_) δ = 7.35–7.18 (7H, *m*, H-14, H-1′, H-5′, H-6′ and H-7′), 5.41 (1H, *br d*, *J* = 11.6 Hz, H-12), 4.98 (1H, *s*, H-17a), 4.91 (1H, *s*, H-17b), 4.84* (1H, *dt*, *J* = 18.5, 1.5 Hz, H-15a), 4.76* (1H, *dt*, *J* = 18.5, 1.7 Hz, H-15b), 4.48 (1H, *dd*, *J* = 11.3, 4.7 Hz, H-3), 4.30 (1H, *d*, *J* = 11.8 Hz, H-19a), 4.11 (2H, *br s*, H-3′), 4.05 (1H, *d*, *J* = 11.8 Hz, H-19b), 2.02 (3H, *s*, H-3OCOCH_3_), 2.01 (3H, *s*, H-19OCOCH_3_), 1.04 (1H, *m*, H-9), 0.95 (3H, *s*, H-18), 0.70 (3H, *s*, H-20) ppm. *AB part of an ABXY spin system with similar coupling values of A (*J* ≅ 1.5) and B (*J* ≅ 1.7) to X and Y. ^13^C-NMR (75 MHz, CDCl_3_) δ = 171.9 (C-16), 170.9 (C-19OCOCH_3_), 170.4 (C-3OCOCH_3_), 147.9 (C-14), 147.7 (C-2′), 145.7 (C-8), 139.0 (C-4′), 133.4 (C-13), 128.7 (C-5′), 128.6 (C-6′), 126.7 (C-7′), 123.0 (C-1′), 108.5 (C-17), 79.5 (C-3), 70.5 (C-15), 64.7 (C-19), 55.7 (C-12), 55.1 (C-5), 51.8 (C-9), 41.2 (C-4), 38.8 (C-10), 38.1 (C-7), 36.8 (C-1), 32.3 (C-3′), 29.7 (C-11), 24.8 (C-6), 24.1 (C-2), 22.3 (C-18), 21.1 (C-3OCOCH_3_), 21.0 (C-19OCOCH_3_), 14.6 (C-20) ppm. ESIMS *m*/*z* 576 [M + H]^+^.
*(12S)-4-benzyl-1,2,3-triazole-3,19-diacetoxy-14-deoxy andrographolide* (**9**): Obtained from reaction of compound **4** (20 mg, 0.044 mmol) with 3-phenyl-1-propyne (6.5 µL, 0.52 mmol). The residue was purified by column chromatography to afford compound **9** (12 mg, 49%) as a white amorphous powder. ^1^H-NMR (300 MHz, CDCl_3_) δ = 7.58 (1H, *br s,* H-14), 7.45 (1H, *s*, H-1′), 7.34–7.18 (5H, *m*, H-5′, H-6′ and H-7′), 5.50 (1H, *dd*, *J* = 11.1, 3.7 Hz, H-12), 4.94 (1H, *s*, H-17a), 4.88 (2H, *br s*, H-15), 4.59 (1H, *s*, H-17b), 4.57 (1H, *dd*, *J* = 11.4, 5.0 Hz, H-3), 4.33 (1H, *d*, *J* = 11.8 Hz, H-19a), 4.08 (1H, *d*, *J* = 11.8 Hz, H-19b), 4.05 (2H, *s*, H-3′), 2.04 (3H, *s*, H-3OCOCH_3_), 2.03 (3H, *s*, H-19OCOCH_3_), 1.52 (1H, *m*, H-9), 0.99 (3H, *s*, H-18), 0.72 (3H, *s*, H-20) ppm. ^13^C-NMR (75 MHz, CDCl_3_) δ = 172.0 (C-16), 170.8 (C-19OCOCH_3_), 170.6 (C-3OCOCH_3_), 150.3 (C-14), 147.5 (C-2′), 145.9 (C-8), 138.8 (C-4′), 131.7 (C-13), 128.7 (C-5′), 128.6 (C-6′), 126.5 (C-7′), 121.2 (C-1′), 108.3 (C-17), 79.6 (C-3), 70.5 (C-15), 64.7 (C-19), 55.2 (C-5 and C-12), 52.4 (C-9), 42.2 (C-4), 39.3 (C-10), 38.1 (C-7), 36.5 (C-1), 32.8 (C-3′), 28.9 (C-11), 24.7 (C-6), 24.1 (C-2), 22.3 (C-18), 21.2 (C-3OCOCH_3_), 21.1 (C-19OCOCH_3_), 14.6 (C-20) ppm. ESIMS *m*/*z* 576 [M + H]^+^.
*(12R)-4-phenyl-1,2,3-triazole-3,19-diacetoxy-14-deoxy-andrographolide* (**10**): Obtained from reaction of compound **3** (30 mg, 0.065 mmol) with phenylacetylene (8.6 µL, 0.78 mmol). The residue was purified by column chromatography to afford compound **10** (11 mg, 37%) as a white amorphous powder. ^1^H-NMR (300 MHz, CDCl_3_) δ = 7.89–7.85 (2H, *m*, H-4′), 7.81 (1H, *s*, H-1′), 7.51–7.30 (3H, *m*, H14, H-5′ and H-6′), 5.54 (1H, *br d*, *J* = 11.8 Hz, H-12), 5.06 (1H, *s*, H-17a), 5.00 (1H, *s*, H-17b), 4.88* (1H, *dt*, *J* = 18.5, 1.5 Hz, H-15a), 4.79* (1H, *dt*, *J* = 18.5, 1.7 Hz, H-15b), 4.48 (1H, *dd*, *J* = 11.6, 4.8 Hz, H-3), 4.31 (1H, *d*, *J* = 11.8 Hz, H-19a), 4.07 (1H, *d*, *J* = 11.8 Hz, H-19b), 2.02 (3H, *s*, H-3OCOCH_3_), 2.00 (3H, *s*, H-19OCOCH_3_), 1.22 (1H, *m*, H-9), 0.92 (3H, *s*, H-18), 0.75 (3H, *s*, H-20) ppm. *AB part of an ABXY spin system with similar coupling values of A (*J* ≅ 1.5) and B (*J* ≅ 1.7) to X and Y. ^13^C-NMR (75 MHz, CDCl_3_) δ = 172.0 (C-16), 170.9 (C-19OCOCH_3_), 170.3 (C-3OCOCH_3_), 147.9 (C-14), 147.5 (C-2′), 145.9 (C-8), 133.6 (C-13), 130.1 (C-3′), 128.9 (C-6′), 128.4 (C-5′), 125.8 (C-4′), 120.5 (C-1′), 108.5 (C-17), 79.4 (C-3), 70.6 (C-15), 64.7 (C-19), 55.9 (C-12), 54.9 (C-5), 51.7 (C-9), 41.2 (C-4), 38.9 (C-10), 38.1 (C-7), 36.3 (C-1), 28.6 (C-11), 25.1 (C-6), 24.1 (C-2), 22.1 (C-18), 21.1 (C-3OCOCH_3_), 21.0 (C-19OCOCH_3_), 14.7 (C-20) ppm. ESIMS *m*/*z* 563 [M + H]^+^.
*(12S)-4-phenyl-1,2,3-triazole-3,19-diacetoxy-14-deoxy-andrographolide* (**11**): Obtained from reaction of compound **4** (30 mg, 0.065 mmol) with phenylacetylene (8.6 µL, 0.78 mmol). The residue was purified by column chromatography to afford compound **11** (11 mg, 31%) as a white amorphous powder. ^1^H-NMR (300 MHz, CDCl_3_) δ = 8.04 (1H, *s*, H-1′), 7.82 (2H, *dd*, *J* = 7.0, 1.7 Hz, H-4′), 7.65 (1H, *br s* H-14), 7.46–7.29 (3H, *m*, H-5′ and H-6′), 5.53 (1H, *dd*, *J* = 11.2, 3.6 Hz, H-12), 5.00 (1H, *s*, H-17a), 4.91 (2H, *br s*, H-15), 4.66 (1H, *s*, H-17b), 4.59 (1H, *dd*, *J* = 11.5, 4.9 Hz, H-3), 4.35 (1H, *d*, *J* = 11.8 Hz, H-19a), 4.10 (1H, *d*, *J* = 11.8 Hz, H-19b), 2.05 (3H, *s*, H-3OCOCH_3_), 2.03 (3H, *s*, H-19OCOCH_3_), 1.59 (1H, *m*, H-9), 1.01 (3H, *s*, H-18), 0.75 (3H, *s*, H-20) ppm. ^13^C-NMR (75 MHz, CDCl_3_) δ = 172.0 (C-16), 170.8 (C-19OCOCH_3_), 170.6 (C-3OCOCH_3_), 150.5 (C-14), 147.9 (C-2′), 145.9 (C-8), 131.5 (C-13), 130.4 (C-3′), 128.3 (C-6′), 128.3 (C-5′), 125.8 (C-4′), 119.4 (C-1′), 108.1 (C-17), 79.6 (C-3), 70.5 (C-15), 64.7 (C-19), 55.4 (C-12), 52.9 (C-5), 51.9 (C-9), 41.2 (C-4), 39.4 (C-10), 38.2 (C-7), 36.9 (C-1), 28.8 (C-11), 24.9 (C-6), 24.2 (C-2), 22.3 (C-18), 21.2 (C-3OCOCH_3_), 21.1 (C-19OCOCH_3_), 14.5 (C-20) ppm. ESIMS *m*/*z* 563 [M + H]^+^.
*(12R)-4-bromophenyl-1,2,3-triazole-3,19-diacetoxy-14-deoxy-andrographolide* (**12**): Obtained from reaction of compound **3** (30 mg, 0.065 mmol) with 2-ethynylpyridine (7.9 µL, 0.78 mmol). The residue was purified by column chromatography to afford compound **12** (35 mg, 83%) as a yellow amorphous powder. ^1^H-NMR (300 MHz, CDCl_3_) δ = 7.83 (1H, *s*, H-1′), 7.72 (2H, *d*, *J* = 8.5 Hz, H-4′), 7.55 (2H, *d*, *J* = 8.5 Hz, H-5′), 7.38 (1H, *q*, *J* = 1.5 Hz, H-14), 5.52 (1H, *br d*, *J* = 11.6 Hz, H-12), 5.03 (1H, *s*, H-17a), 4.94 (1H, *s*, H-17b), 4.87* (1H, *dt*, *J* = 18.5, 1.5 Hz, H-15a), 4.78* (1H, *dt*, *J* = 18.5, 1.7 Hz, H-15b), 4.46 (1H, *dd*, *J* = 11.5, 4.9 Hz, H-3), 4.29 (1H, *d*, *J* = 11.7 Hz, H-19a), 4.04 (1H, *d*, *J* = 11.7 Hz, H-19b), 2.00 (3H, *s*, H-3OCOCH_3_), 1.98 (3H, *s*, H-19OCOCH_3_), 1.15 (1H, *m*, H9), 0.90 (3H, *s*, H-18), 0.73 (3H, *s*, H-20) ppm. *AB part of an ABXY spin system with similar coupling values of A (*J* ≅ 1.5) and B (*J* ≅ 1.7) to X and Y. ^13^C-NMR (75 MHz, CDCl_3_) δ = 171.9 (C-16), 170.9 (C-19OCOCH_3_), 170.4 (C-3OCOCH_3_), 148.1 (C-14), 146.5 (C-2′), 145.8 (C-8), 133.3 (C-13), 132.2 (C-5′), 129.1 (C-3′), 127.3 (C-4′), 122.3 (C-6′), 120.6 (C-1′), 108.5 (C-17), 79.4 (C-3), 70.6 (C-15), 64.7 (C-19), 55.9 (C-12), 54.9 (C-5), 51.9 (C-9), 41.2 (C-4), 39.0 (C-10), 38.2 (C-7), 36.4 (C-1), 28.7 (C-11), 24.8 (C-6), 24.1 (C-2), 22.2 (C-18), 21.1 (C-3OCOCH_3_), 21.0 (C-19OCOCH_3_), 14.7 (C-20) ppm. ESIMS *m*/*z* 564 [M + H]^+^.
*(12R)-4-aniline-1,2,3-triazole-3,19-diacetoxy-14-deoxy-andrographolide* (**13**): Obtained from reaction of compound **3** (30 mg, 0.065 mmol) with 4-ethynylaniline (9.8 µL, 0.78 mmol). The residue was purified by column chromatography to afford compound **13** (3 mg, 8%) as a white amorphous powder. ^1^H-NMR (300 MHz, CDCl_3_) δ = 7.66 (2H, *d*, *J* = 8.4 Hz, H-4′), 7.35 (1H, *q*, *J* = 1,5 Hz H-14), 6.75 (2H, *d*, *J* = 8.4 Hz, H-5′), 5.50 (1H, *br d*, *J* = 11.8 Hz, H-12), 5.05 (1H, *s*, H-17a), 5.01 (1H, *s*, H-17b), 4.87* (1H, *dt*, *J* = 18.5, 1.5 Hz, H-15a), 4.74* (1H, *dt*, *J* = 18.5, 1.7 Hz, H-15b), 4.48 (1H, *dd*, *J* = 11.7, 4.8 Hz, H-3), 4.31 (1H, *d*, *J* = 11.8 Hz, H-19a), 4.07 (1H, *d*, *J* = 11.8 Hz, H-19b), 2.02 (3H, *s*, H-3OCOCH_3_), 2.00 (3H, *s*, H-19OCOCH_3_), 0.93 (3H, *s*, H-18), 0.75 (3H, *s*, H-20) ppm. *AB part of an ABXY spin system with similar coupling values of A (*J* ≅ 1.5) and B (*J* ≅ 1.7) to X and Y. ESIMS *m*/*z* 577 [M + H]^+^.
*(12R)-4-benzonitrile-1,2,3-triazole-3,19-diacetoxy-14-deoxy-andrographolide* (**14**): Obtained from reaction of compound **3** (30 mg, 0.065 mmol) with 4-ethynylbenzonitrile (10.7 µL, 0.78 mmol). The residue was purified by column chromatography to afford compound **14** (18 mg, 47%) as a white amorphous powder. ^1^H-NMR (300 MHz, CDCl_3_) δ = 7.98 (2H, *d*, *J* = 8.5 Hz, H-4′), 7.96 (1H, *s*, H-1′), 7.73 (2H, *d*, *J* = 8.5 Hz, H-5′), 7.44 (1H, *q J* = 1.5 Hz, H-14), 5.56 (1H, *br d*, *J* = 11.6 Hz, H-12), 5.05 (1H, *s*, H-17a), 4.92 (1H, *s*, H-17b), 4.89* (1H, *dt*, *J* = 18.5, 1.5 Hz, H-15a), 4.81* (1H, *dt*, *J* = 18.5, 1.7 Hz, H-15b), 4.46 (1H, *dd*, *J* = 11.4, 5.0 Hz, H-3), 4.30 (1H, *d*, *J* = 11.8 Hz, H-19a), 4.06 (1H, *d*, *J* = 11.8 Hz, H-19b), 2.01 (3H, *s*, H-3OCOCH_3_), 2.00 (3H, *s*, H-19OCOCH_3_), 1.14 (1H, *m*, H-9), 0.91 (3H, *s*, H-18), 0.75 (3H, *s*, H-20) ppm. *AB part of an ABXY spin system with similar coupling values of A (*J* ≅ 1.5) and B (*J* ≅ 1.7) to X and Y. ^13^C-NMR (75 MHz, CDCl_3_) δ = 171.9 (C-16), 170.9 (C-19OCOCH_3_), 170.4 (C-3OCOCH_3_), 149.0 (C-2′), 148.2 (C-14), 145.8 (C-8), 134.6 (C-3′), 133.1 (C-13), 132.8 (C-5′), 126.2 (C-4′), 121.7 (C-1′), 118.7 (C-7′), 111.8 (C-6′), 108.5 (C-17), 79.4 (C-3), 70.6 (C-15), 64.7 (C-19), 56.0 (C-12), 55.0 (C-5), 52.0 (C-9), 41.2 (C-4), 39.0 (C-10), 38.2 (C-7), 36.5 (C-1), 28.7 (C-11), 24.8 (C-6), 24.1 (C-2), 22.2 (C-18), 21.1 (C-3OCOCH_3_), 21.0 (C-19OCOCH_3_), 14.7 (C-20) ppm. ESIMS *m*/*z* 587 [M + H]^+^.
*(12S)-4-benzonitrile-1,2,3-triazole-3,19-diacetoxy-14-deoxy-andrographolide* (**15**): Obtained from reaction of compound **4** (20 mg, 0.044 mmol) with 4-ethynylbenzonitrile (7.1 µL, 0.52 mmol). The residue was purified by column chromatography to afford compound **15** (9 mg, 36%) as a white amorphous powder. ^1^H-NMR (300 MHz, CDCl_3_) δ = 8.19 (1H, *s*, H-1′), 7.94 (2H, *d*, *J* = 8.6 Hz, H-4′), 7.70 (2H, *d*, *J* = 8.6 Hz, H-5′), 7.67 (1H, *br s*, Hz, H-14), 5.67 (1H, *dd*, *J* = 11.4, 3.5 Hz, H-12), 5.01 (1H, *s*, H-17a), 4.93 (2H, *br s*, H-15), 4.65 (1H, *s*, H-17b), 4.59 (1H, *dd*, *J* = 11.6, 4.9 Hz, H-3), 4.35 (1H, *d*, *J* = 11.8 Hz, H-19a), 4.10 (1H, *d*, *J* = 11.8 Hz, H-19b), 2.05 (3H, *s*, H-3OCOCH_3_), 2.03 (3H, *s*, H-19OCOCH_3_), 1.50 (1H, *m*, H-9), 1.01 (3H, *s*, H-18), 0.75 (3H, *s*, H-20) ppm. ^13^C-NMR (75 MHz, CDCl_3_) δ = 171.9 (C-16), 170.8 (C-19OCOCH_3_), 170.6 (C-3OCOCH_3_), 150.9 (C-14), 146.1 (C-2′), 145.8 (C-8), 134.8 (C-13), 132.7 (C-5′), 131.0 (C-3′), 126.1 (C-4′), 120.6 (C-1′), 118.7 (C-7′), 108.1 (C-17), 79.6 (C-3), 70.5 (C-15), 64.6 (C-19), 55.7 (C-12), 53.0 (C-5), 53.0 (C-9), 41.3 (C-4), 39.4 (C-10), 38.3 (C-7), 37.0 (C-1), 29.4 (C-11), 24.8 (C-6), 24.2 (C-2), 22.4 (C-18), 21.2 (C-3OCOCH_3_), 21.1 (C-19OCOCH_3_), 14.6 (C-20) ppm. ESIMS *m*/*z* 587 [M + H]^+^.
*(12R)-4-(4-methoxyphenyl)-1,2,3-triazole-3,19-diacetoxy-14-deoxy-andrographolide* (**16**): Obtained from reaction of compound **3** (30 mg, 0.065 mmol) with 4-ethynylanisole (10.2 µL, 0.78 mmol). The residue was purified by column chromatography to afford compound **16** (17 mg, 43%) as a white amorphous powder. ^1^H-NMR (300 MHz, CDCl_3_) δ = 7.79 (2H, *d*, *J* = 8.8 Hz, H-4′), 7.72 (1H, *s*, H-1′), 7.36 (2H, *d*, *J* = 8.8 Hz, H-5′), 7.36 (1H, *q*, *J* = 1.6 Hz, H-14), 5.52 (1H, *br d*, *J* = 11.5 Hz, H-12), 5.05 (1H, *s*, H-17a), 4.99 (1H, *s*, H-17b), 4.87* (1H, *dt*, *J* = 18.5, 1.5 Hz, H-15a), 4.78* (1H, *dt*, *J* = 18.5, 1.7 Hz, H-15b), 4.47 (1H, *dd*, *J* = 11.6, 4.8 Hz, H-3), 4.31 (1H, *d*, *J* = 11.7 Hz, H-19a), 4.06 (1H, *d*, *J* = 11.7 Hz, H-19b), 3.84 (3H, *s*, H-7′), 2.01 (3H, *s*, H-19OCOCH_3_), 1.99 (3H, *s*, H-3OCOCH_3_), 1.24 (1H, *m*, H-9), 0.92 (3H, *s*, H-18), 0.74 (3H, *s*, H-20) ppm. *AB part of an ABXY spin system with similar coupling values of A (*J* ≅ 1.5) and B (*J* ≅ 1.7) to X and Y. ^13^C-NMR (75 MHz, CDCl_3_) δ = 172.0 (C-16), 170.9 (C-19OCOCH_3_), 170.3 (C-3OCOCH_3_), 159.8 (C-6′), 147.7 (C-14), 147.7 (C-2′), 145.9 (C-8), 133.6 (C-13), 127.1 (C-4′), 122.9 (C-3′), 119.6 (C-1′), 114.4 (C-5′), 108.6 (C-17), 79.4 (C-3), 70.5 (C-15), 64.7 (C-19), 55.8 (C-12), 55.4 (C-7′), 54.9 (C-5), 51.8 (C-9), 41.2 (C-4), 39.0 (C-10), 38.2 (C-7), 36.4 (C-1), 28.2 (C-11), 24.8 (C-6), 24.1 (C-2), 22.3 (C-18), 21.1 (C-3OCOCH_3_), 21.0 (C-19OCOCH_3_), 14.7 (C20) ppm. ESIMS *m/z* 592 [M + H]^+^.
*(12S)-4-(4-methoxyphenyl)-1,2,3-triazole-3,19-diacetoxy-14-deoxy-andrographolide* (**17**): Obtained from reaction of compound **4** (30 mg, 0.065 mmol) with 4-ethynylanisole (10.2 µL, 0.78 mmol). The residue was purified by column chromatography to afford compound **17** (7 mg, 18%) as a white amorphous powder. ^1^H-NMR (300 MHz, CDCl_3_) δ = 7.95 (1H, *s*, H-1′), 7.74 (2H, *d*, *J* = 8.7 Hz, H-4′), 7.64 (1H, *br s*, H-14), 6.95 (2H, *d*, *J* = 8.7 Hz, H-5′), 5.62 (1H, *dd*, *J* = 11.2, 3.6 Hz, H-12), 5.00 (1H, *s*, H-17a), 4.91 (2H, *br s*, H-15), 4.66 (1H, *s*, H-17b), 4.59 (1H, *dd*, *J* = 11.5, 4.9 Hz, H-3), 4.35 (1H, *d*, *J* = 11.8 Hz, H-19a), 4.10 (1H, *d*, *J* = 11.8 Hz, H-19b), 3.84 (3H, *s*, H-7′), 2.05 (3H, *s*, H-3OCOCH_3_), 2.03 (3H, *s*, H-19OCOCH_3_), 1.52 (1H, *m*, H-9), 1.01 (3H, *s*, H-18), 0.75 (3H, *s*, H-20) ppm. ^13^C-NMR (75 MHz, CDCl_3_) δ = 172.0 (C-16), 170.8 (C-19OCOCH_3_), 170.6 (C-3OCOCH_3_), 159.7 (C-6′), 150.5 (C-14), 147.7 (C-2′), 145.9 (C-8), 131.6 (C-13), 127.1 (C-4′), 123.1 (C-3′), 118.6 (C-1′), 114.2 (C-5′), 108.1 (C-17), 79.6 (C-3), 70.5 (C-15), 64.7 (C-19), 55.4 (C-12), 55.3 (C-7′), 52.9 (C-5), 51.8 (C-9), 41.3 (C-4), 39.3 (C-10), 38.2 (C-7), 36.5 (C-1), 29.4 (C-11), 23.9 (C-6), 24.2 (C-2), 22.2 (C-18), 21.2 (C-3OCOCH_3_), 21.1 (C-19OCOCH_3_), 14.3 (C-20) ppm. ESIMS *m/z* 592 [M + H]^+^.
*(12R)-4-(4-trifluoromethoxy)phenyl)-1,2,3-triazole-3,19-diacetoxy-14-deoxy-andrographolide* (**18**): Obtained from reaction of compound **3** (30 mg, 0.065 mmol) with 4-(trifluoromethoxy)phenylacetylene (12 µL, 0.78 mmol). The residue was purified by column chromatography to afford compound **18** (22 mg, 51%) as a white amorphous powder. ^1^H-NMR (300 MHz, CDCl_3_) δ = 7.89 (2H, *dm*, *J* = 8.8 Hz, H-4′), 7.84 (1H, *s*, H-1′), 7.41 (1H, *q*, *J* = 1.6 Hz, H-14), 7.29 (2H, *dd*, *J* = 8.8 Hz, H-5′), 5.54 (1H, *br d*, *J* = 11.7 Hz, H-12), 5.06 (1H, *s*, H-17a), 4.96 (1H, *s*, H-17b), 4.88* (1H, *dt*, *J* = 18.5, 1.4 Hz, H-15a), 4.80* (1H, *dt*, *J* = 18.5, 1.7 Hz, H-15b), 4.47 (1H, *dd*, *J* = 11.5, 4.9 Hz, H-3), 4.30 (1H, *d*, *J* = 11.7 Hz, H-19a), 4.06 (1H, *d*, *J* = 11.7 Hz, H-19b), 2.01 (3H, *s*, H-3OCOCH_3_), 2.00 (3H, *s*, H-19OCOCH_3_), 1.10 (1H, *m*, H-9), 0.92 (3H, *s*, H-18), 0.75 (3H, *s*, H-20) ppm. *AB part of an ABXY spin system with similar coupling values of A (*J* ≅ 1.5) and B (*J* ≅ 1.7) to X and Y. ^13^C-NMR (75 MHz, CDCl_3_) δ = 171.9 (C-16), 170.8 (C-19OCOCH_3_), 170.4 (C-3OCOCH_3_), 149.2 (C-6′), 148.0 (C-14), 146.3 (C-2′), 145.8 (C-8), 133.4 (C-13), 129.0 (C-3′), 127.2 (C-4′), 121.5 (C-5’), 120.6 (C-1’), 108.5 (C-17), 79.4 (C-3), 70.6 (C-15), 64.7 (C-19), 55.9 (C-12), 55.0 (C-5), 51.9 (C-9), 41.2 (C-4), 39.0 (C-10), 38.2 (C-7), 36.5 (C-1), 28.8 (C-11), 24.8 (C-6), 24.1 (C-2), 22.5 (C-18), 21.1 (C-3OCOCH_3_), 21.0 (C-19OCOCH_3_), 14.7 (C-20) ppm. ESIMS *m*/*z* 646 [M + H]^+^.
*(12S)-4-(4-trifluoromethoxy)phenyl)-1,2,3-triazole-3,19-diacetoxy-14-deoxy-andrographolide* (**19**): Obtained from reaction of compound **4** (30 mg, 0.065 mmol) with 4-(trifluoromethoxy)phenylacetylene (12 µL, 0.78 mmol). The residue was purified by column chromatography to afford compound **19** (11 mg, 25%) as a white amorphous powder. ^1^H-NMR (300 MHz, CDCl_3_) δ = 8.06 (1H, *s*, H-1′), 7.85 (2H, *dd*, *J* = 8.8 Hz, H-4′), 7.65 (1H, *br s*, H-14), 7.26 (2H, *dm*, *J* = 8.8 Hz, H-5′), 5.65 (1H, *dd*, *J* = 11.1, 3.5 Hz, H-12), 5.00 (1H, *s*, H-17a), 4.92 (2H, *br s*, H-15), 4.66 (1H, *s*, H-17b), 4.59 (1H, *dd*, *J* = 11.6, 4.9 Hz, H-3), 4.35 (1H, *d*, *J* = 11.8 Hz, H-19a), 4.10 (1H, *d*, *J* = 11.8 Hz, H-19b), 2.05 (3H, *s*, H-3OCOCH_3_), 2.03 (3H, *s*, H-19OCOCH_3_), 1.53 (1H, *m*, H-9), 1.01 (3H, *s*, H-18), 0.75 (3H, *s*, H-20) ppm. ^13^C-NMR (75 MHz, CDCl_3_) δ = 171.9 (C-16), 170.8 (C-19OCOCH_3_), 170.6 (C-3OCOCH_3_), 150.7 (C-14), 149.1 (C-6′), 146.6 (C-2′), 145.9 (C-8), 131.3 (C-13), 129.2 (C-3′), 127.2 (C-4′), 121.3 (C-5′), 119.6 (C-1′), 108.1 (C-17), 79.6 (C-3), 70.5 (C-15), 64.6 (C-19), 55.5 (C-12), 55.3 (C-5), 52.9 (C-9), 41.3 (C-4), 39.4 (C-10), 38.3 (C-7), 36.9 (C-1), 28.7 (C-11), 24.8 (C-6), 24.2 (C-2), 22.6 (C-18), 21.2 (C-3OCOCH_3_), 21.1 (C-19OCOCH_3_), 14.6 (C-20) ppm. ESIMS *m*/*z* 646 [M + H]^+^.
*(12R)-4-(3-biphenyl)-1,2,3-triazole-3,19-diacetoxy-14-deoxy-andrographolide* (**20**): Obtained from reaction of compound **3** (30 mg, 0.065 mmol) with 3-ethynylbiphenyl (14.0 mg, 0.78 mmol). The residue was purified by column chromatography to afford compound **20** (32 mg, 76%) as a white amorphous powder. ^1^H-NMR (300 MHz, CDCl_3_) δ = 7.94 (2H, *d*, *J* = 8.4 Hz, H-4′), 7.86 (1H, *s*, H-1′), 7.68 (2H, *d*, *J* = 8.4 Hz, H-5′), 7.63 (2H, *br d*, *J* = 7.2 Hz, H-8′), 7.45 (2H, *t*, *J* = 7.2 Hz, H-9′), 7.40 (1H, *q*, *J* = 1.5 Hz, H-14), 7.38–7.32 (1H, *m*, H-10′), 5.55 (1H, *br d*, *J* = 11.4 Hz, H-12), 5.06 (1H, *s*, H-17a), 5.00 (1H, *s*, H-17b), 4.88* (1H, *dt*, *J* = 18.5, 1.5 Hz, H-15a), 4.79* (1H, *dt*, *J* = 18.5, 1.7 Hz, H-15b), 4.48 (1H, *dd*, *J* = 11.6, 4.8 Hz, H-3), 4.31 (1H, *d*, *J* = 11.8 Hz, H-19a), 4.06 (1H, *d*, *J* = 11.8 Hz, H-19b), 2.01 (3H, *s*, H-3OCOCH_3_), 1.99 (3H, *s*, H-19OCOCH_3_), 1.25 (1H, *m*, H-9), 0.92 (3H, *s*, H-18), 0.75 (3H, *s*, H-20) ppm. *AB part of an ABXY spin system with similar coupling values of A (*J* ≅ 1.5) and B (*J* ≅ 1.7) to X and Y. ^13^C-NMR (75 MHz, CDCl_3_) δ = 172.0 (C-16), 170.9 (C-19OCOCH_3_), 170.3 (C-3OCOCH_3_), 148.0 (C-14), 147.2 (C-2′), 145.9 (C-8), 141.2 (C-6′), 140.5 (C-7′), 133.5 (C-13), 129.1 (C-3′), 128.8 (C-9′), 127.6 (C-10′), 127.5 (C-5′), 127.0 (C-8′), 126.2 (C-4′), 120.5 (C-1′), 108.5 (C-17), 79.4 (C-3), 70.6 (C-15), 64.7 (C-19), 55.9 (C-12), 54.9 (C-5), 51.9 (C-9), 41.2 (C-4), 39.0 (C-10), 38.2 (C-7), 36.4 (C-1), 28.9 (C-11), 24.8 (C-6), 24.1 (C-2), 22.5 (C-18), 21.1 (C-3OCOCH_3_), 21.0 (C-19OCOCH_3_), 14.7 (C-20) ppm. ESIMS *m*/*z* 638 [M + H]^+^.
*(12S)-4-(3-biphenyl)-1,2,3-triazole-3,19-diacetoxy-14-deoxy-andrographolide* (**21**): Obtained from reaction of compound **4** (20 mg, 0.044 mmol) with 3-ethynylbiphenyl (9.3 mg, 0.52 mmol). The residue was purified by column chromatography to afford compound **21** (12 mg, 44%) as a white amorphous powder. ^1^H-NMR (300 MHz, CDCl_3_) δ = 8.08 (1H, *s*, H-1′), 7.89 (2H, *d*, *J* = 8.4, Hz, H-4′), 7.66 (2H, *d*, *J* = 8.4 Hz, H-5′), 7.66 (1H, *s*, H-14), 7.62 (2H, *d*, *J* = 7.3 Hz, H-8′), 7.45 (2H, *t*, *J* = 7.3 Hz, H-9′), 7.39–7.31 (1H, *m*, H-10′), 5.65 (1H, *dd*, *J* = 11.2, 3.6 Hz, H-12), 5.01 (1H, *s*, H-17a), 4.92 (2H, *br s*, H-15), 4.68 (1H, *s*, H-17b), 4.59 (1H, *dd*, *J* = 11.6, 4.9 Hz, H-3), 4.35 (1H, *d*, *J* = 11.8 Hz, H-19a), 4.11 (1H, *d*, *J* = 11.8 Hz, H-19b), 2.06 (3H, *s*, H-3OCOCH_3_), 2.04 (3H, *s*, H-19OCOCH_3_), 1.59 (1H, *m*, H-9), 1.01 (3H, *s*, H-18), 0.76 (3H, *s*, H-20) ppm. ^13^C-NMR (75 MHz, CDCl_3_) δ = 172.0 (C-16), 170.8 (C-19OCOCH_3_), 170.6 (C-3OCOCH_3_), 150.6 (C-14), 147.6 (C-2′), 145.9 (C-8), 141.0 (C-6′), 140.6 (C-7′), 131.5 (C-13), 129.3 (C-3′), 128.8 (C-9′), 127.5 (C-10′), 127.4 (C-5′), 127.0 (C-8′), 126.1 (C-4′), 119.4 (C-1′), 108.1 (C-17), 79.6 (C-3), 70.5 (C-15), 64.7 (C-19), 55.5 (C-12), 55.4 (C-5), 53.0 (C-9), 41.3 (C-4), 39.9 (C-10), 38.2 (C-7), 36.6 (C-1), 29.0 (C-11), 24.8 (C-6), 24.3 (C-2), 22.6 (C-18), 21.2 (C-3OCOCH_3_), 21.1 (C-19OCOCH_3_), 14.6 (C-20) ppm. ESIMS *m/z* 638 [M + H]^+^.
*(12R)-4-(4-pyridine)-1,2,3-triazole-3,19-diacetoxy-14-deoxy-andrographolide* (**22**): Obtained from reaction of compound **3** (30 mg, 0.065 mmol) with 3-ethynylthiophene (8.1 mg, 0.78 mmol). The residue was purified by column chromatography to afford compound **22** (16 mg, 44%) as a white amorphous powder. ^1^H-NMR (300 MHz, CDCl_3_) δ = 8.68 (2H, *br s*, H-5′), 7.99 (1H, *s*, H-1′), 7.75 (2H, *d*, *J* = 5.7 Hz, H-4′), 7.43 (1H, *q*, *J* = 1.5 Hz, H-14), 5.56 (1H, *br d*, *J* = 11.5 Hz, H-12), 5.06 (1H, *s*, H-17a), 4.93 (1H, *s*, H-17b), 4.89* (1H, *dt*, *J* = 18.5, 1.5 Hz, H-15a), 4.80* (1H, *dt*, *J* = 18.5, 1.7 Hz, H-15b), 4.47 (1H, *dd*, *J* = 11.5, 4.9 Hz, H-3), 4.30 (1H, *d*, *J* = 11.8 Hz, H-19a), 4.06 (1H, *d*, *J* = 11.8 Hz, H-19b), 2.01 (3H, *s*, H-3OCOCH_3_), 1.98 (3H, *s*, H-19OCOCH_3_), 1.14 (1H, *m*, H-9), 0.91 (3H, *s*, H-18), 0.75 (3H, *s*, H-20) ppm. *AB part of an ABXY spin system with similar coupling values of A (*J* ≅ 1.5) and B (*J* ≅ 1.7) to X and Y. ^13^C-NMR (75 MHz, CDCl_3_) δ = 171.9 (C-16), 170.9 (C-19OCOCH_3_), 170.4 (C-3OCOCH_3_), 150.5 (C-5′), 148.1 (C-14), 145.8 (C-8), 145.1 (C-2′), 137.6 (C-3′), 133.1 (C-13), 122.1 (C-4′), 120.0 (C-1′), 108.5 (C-17), 79.4 (C-3), 70.6 (C-15), 64.7 (C-19), 56.0 (C-12), 55.0 (C-5), 51.9 (C-9), 42.2 (C-4), 39.0 (C-10), 38.2 (C-7), 36.4 (C-1), 28.8 (C-11), 24.8 (C-6), 24.1 (C-2), 22.2 (C-18), 21.1 (C-3OCOCH_3_), 21.0 (C-19OCOCH_3_), 14.7 (C-20) ppm. ESIMS *m*/*z* 563 [M + H]^+^.
*(12S)-4-(4-pyridine)-1,2,3-triazole-3,19-diacetoxy-14-deoxy-andrographolide* (**23**): Obtained from reaction of compound **4** (20 mg, 0.044 mmol) with 3-ethynylthiophene (5.4 mg, 0.52 mmol). The residue was purified by column chromatography to afford compound **23** (5 mg, 21%) as a white amorphous powder. ^1^H-NMR (300 MHz, CDCl_3_) δ = 8.66 (1H, *br s*, H-5′), 7.72 (2H, *d*, *J* = 5.1 Hz, H-4′), 7.64 (1H, *br s*, H-14), 5.68 (1H, *dd*, *J* = 11.5, 3.4 Hz, H-12), 5.02 (1H, *s*, H-17a), 4.94 (2H, *br s*, H-15), 4.66 (1H, *s*, H-17b), 4.59 (1H, *dd*, *J* = 11.6, 4.9 Hz, H-3), 4.35 (1H, *d*, *J* = 11.8 Hz, H-19a), 4.11 (1H, *d*, *J* = 11.8 Hz, H-19b), 2.05 (3H, *s*, H-3OCOCH_3_), 2.04 (3H, *s*, H-19OCOCH_3_), 1.02 (3H, *s*, H-18), 0.76 (3H, *s*, H-20) ppm. ESIMS *m*/*z* 563 [M + H]^+^.
*(12R)-4-pyridine-1,2,3-triazole-3,19-diacetoxy-14-deoxy-andrographolide* (**24**): Obtained from reaction of compound **3** (30 mg, 0.065 mmol) with 2-ethynylpyridine (7.9 µL, 0.78 mmol). The residue was purified by column chromatography to afford compound **24** (32 mg, 88%) as a white amorphous powder. ^1^H-NMR (300 MHz, CDCl_3_) δ = 8.58 (1H, *ddd*, *J* = 4.9, 1.8, 0.9 Hz, H-7′), 8.21 (1H, *dt*, *J* = 7.8, 1.2 Hz, H-4′), 8.16 (1H, *s*, H-1′), 7.80 (1H, *td*, *J* = 7.8, 1.8 Hz, H-5′), 7.29 (1H, *q*, *J* = 1.6 Hz, H-14), 7.24 (1H, *ddd*, *J* = 7.5, 4.9, 1.2 Hz, H-6′), 5.56 (1H, *br d*, *J* = 11.5 Hz, H-12), 5.04 (1H, *s*, H-17a), 5.03 (1H, *s*, H-17b), 4.86* (1H, *dt*, *J* = 18.5, 1.5 Hz, H-15a), 4.76* (1H, *dt*, *J* = 18.5, 1.7 Hz, H-15b), 4.45 (1H, *dd*, *J* = 11.4, 5.0 Hz, H-3), 4.30 (1H, *d*, *J* = 11.8 Hz, H-19a), 4.04 (1H, *d*, *J* = 11.8 Hz, H-19b), 2.00 (3H, *s*, H-3OCOCH_3_), 1.98 (3H, *s*, H-19OCOCH_3_), 1.17 (1H, *m*, H-9), 0.90 (3H, *s*, H-18), 0.73 (3H, *s*, H-20) ppm. *AB part of an ABXY spin system with similar coupling values of A (*J* ≅ 1.5) and B (*J* ≅ 1.7) to X and Y. ^13^C-NMR (75 MHz, CDCl_3_) δ = 171.7 (C-16), 170.8 (C-19OCOCH_3_), 170.4 (C-3OCOCH_3_), 149.9 (C-3′), 149.5 (C-7′), 148.2 (C-2′), 147.6 (C-14), 145.6 (C-8), 137.0 (C-5′), 133.6 (C-13), 123.1 (C-6′), 122.8 (C-1′), 120.4 (C-4′), 108.7 (C-17), 79.5 (C-3), 70.5 (C-15), 64.7 (C-19), 55.3 (C-12), 54.9 (C-5), 51.9 (C-9), 41.2 (C-4), 38.9 (C-10), 38.1 (C-7), 36.4 (C-1), 28.9 (C-11), 24.8 (C-6), 24.1 (C-2), 22.5 (C-18), 21.1 (C-3OCOCH_3_), 21.0 (C-19OCOCH_3_), 14.7 (C-20) ppm. ESIMS *m*/*z* 564 [M + H]^+^.
*(12R)-4-(3-thiophene)-1,2,3-triazole-3,19-diacetoxy-14-deoxy-andrographolide* (**25**): Obtained from reaction of compound **3** (30 mg, 0.065 mmol) with 3-ethynylthiophene (7.8 µL, 0.78 mmol). The residue was purified by column chromatography to afford compound **25** (30 mg, 82%) as a white amorphous powder. ^1^H-NMR (300 MHz, CDCl_3_) δ = 7.72 (1H, *dd*, *J* = 3.0, 1.3 Hz, H-6′), 7.71 (1H, *s*, H-1′), 7.47 (1H, *dd*, *J* = 5.0, 3.0 Hz, H-4′), 7.39 (1H, *dd*, *J* = 5.0, 1.3 Hz, H-5′), 7.35 (1H, *q*, *J* = 1.6 Hz, H-14), 5.50 (1H, *br d*, *J* = 11.5 Hz, H-12), 5.04 (1H, *s*, H-17a), 4.97 (1H, *s*, H-17b), 4.86* (1H, *dt*, *J* = 18.5, 1.5 Hz, H-15a), 4.77* (1H, *dt*, *J* = 18.5, 1.7 Hz, H-15b), 4.46 (1H, *dd*, *J* = 11.6, 4.8 Hz, H-3), 4.30 (1H, *d*, *J* = 11.7 Hz, H-19a), 4.05 (1H, *d*, *J* = 11.7 Hz, H-19b), 2.00 (3H, *s*, H-3OCOCH_3_), 1.99 (3H, *s*, H-19OCOCH_3_), 1.17 (1H, *m*, H-9), 0.91 (3H, *s*, H-18), 0.74 (3H, *s*, H-20) ppm. *AB part of an ABXY spin system with similar coupling values of A (*J* ≅ 1.5) and B (*J* ≅ 1.7) to X and Y. ^13^C-NMR (75 MHz, CDCl_3_) δ = 171.4 (C-16), 170.9 (C-19OCOCH_3_), 170.3 (C-3OCOCH_3_), 147.9 (C-14), 145.9 (C-8), 143.8 (C-2′), 133.5 (C-13), 131.4 (C-3′), 126.5 (C-5′), 125.7 (C-4′), 121.5 (C-6′), 120.3 (C-1′), 108.5 (C-17), 79.4 (C-3), 70.6 (C-15), 64.7 (C-19), 55.8 (C-12), 54.9 (C-5), 51.8 (C-9), 41.2 (C-4), 39.0 (C-10), 38.2 (C-7), 36.4 (C-1), 28.8 (C-11), 24.8 (C-6), 24.1 (C-2), 22.2 (C-18), 21.1 (C-3OCOCH_3_), 21.0 (C-19OCOCH_3_), 14.7 (C-20) ppm. ESIMS *m*/*z* 568 [M + H]^+^.
*(12S)-4-(3-thiophene)-1,2,3-triazole-3,19-diacetoxy-14-deoxy-andrographolide* (**26**): Obtained from reaction of compound **4** (20 mg, 0.044 mmol) with 3-ethynylthiophene (5.2 µL, 0.52 mmol). The residue was purified by column chromatography to afford compound **26** (5 mg, 21%) as a white amorphous powder. ^1^H-NMR (300 MHz, CDCl_3_) δ = 8.66 (1H, *s*, H-6′), 8.22 (1H, *s*, H-1′), 7.75–7.70 (2H, *m*, H-4′ and H-5′), 7.67 (1H, *br s*, H-14), 5.68 (1H, *dd*, *J* = 11.4, 3.5 Hz, H-12), 5.02 (1H, *s*, H-17a), 4.94 (2H, *br s*, H-15), 4.66 (1H, *s*, H-17b), 4.59 (1H, *dd*, *J* = 11.6, 4.9 Hz, H-3), 4.35 (1H, *d*, *J* = 11.8 Hz, H-19a), 4.11 (1H, *d*, *J* = 11.8 Hz, H-19b), 2.05 (3H, *s*, H-3OCOCH_3_), 2.04 (3H, *s*, H-19OCOCH_3_), 1.60 (1H, *m*, H-9), 1.02 (3H, *s*, H-18), 0.76 (3H, *s*, H-20) ppm. ^13^C-NMR (75 MHz, CDCl_3_) δ = 172.0 (C-16), 170.8 (C-19OCOCH_3_), 170.6 (C-3OCOCH_3_), 150.5 (C-14), 146.0 (C-8), 144.0 (C-2′), 131.6 (C-13), 131.5 (C-3′), 126.3 (C-5′), 125.8 (C-4′), 121.3 (C-6′), 119.2 (C-1′), 108.1 (C-17), 79.6 (C-3), 70.5 (C-15), 64.6 (C-19), 55.7 (C-12), 55.4 (C-5), 52.9 (C-9), 41.3 (C-4), 39.3 (C-10), 38.2 (C-7), 36.4 (C-1), 28.5 (C-11), 24.8 (C-6), 24.2 (C-2), 22.3 (C-18), 21.2 (C-3OCOCH_3_), 21.1 (C-19OCOCH_3_), 14.7 (C20) ppm. ESIMS *m*/*z* 568 [M + H]^+^.

### 3.2. Biology

#### 3.2.1. Human Cell Lines and Growth Conditions

Human breast adenocarcinoma MCF-7, pancreatic ductal adenocarcinoma PANC-1, melanoma A375, and non-tumorigenic colon CCD-18Co cell lines were purchased from ATCC (Rockville, MD, USA). Human colorectal adenocarcinoma HCT116 cells were kindly provided by B. Vogelstein (The Johns Hopkins Kimmel Cancer Center, Baltimore, MD, USA). Tumor cells were routinely cultured in RPMI-1640 medium with UltraGlutamine from Biowest (VWR, Carnaxide, Portugal) supplemented with 10% fetal bovine serum (FBS) from Biowest (VWR, Carnaxide, Portugal), except for the PANC-1 cells, which were grown in DMEM (4.5 g/L glucose) with stable L-glutamine and sodium pyruvate and supplemented with 10% FBS. The non-tumorigenic CCD-18Co cells were cultured in EMEM (Lonza, VWR, Carnaxide, Portugal), supplemented with 10% FBS. All cells were incubated at 37 °C in a humidified atmosphere of 5% CO_2_. Routine mycoplasma testing was performed using the MycoAlert™ PLUS mycoplasma detection kit (Lonza).

#### 3.2.2. Sulforhodamine B (SRB) Assay

For the evaluation of the compound effect on cell proliferation, human cell lines were seeded in 96-well plates at a density of 5.0 × 10^3^ (HCT116, MCF-7, PANC-1, A375, and CCD-18Co) cells per well, for 24 h. Cells were then treated with serial dilutions of the compound (ranging from 0.63 to 30 µM), for an additional 48 h incubation period. The growth inhibitory effects were assessed by SRB assay, as described in [58], and the half-maximal inhibitory concentration (IC_50_ values) were determined for each cell line using the GraphPad Prism software version 7.0 (La Jolla, CA, USA). Culture medium containing a maximum of 0.05% dimethyl sulfoxide (DMSO) was used as negative control.

#### 3.2.3. Cell Cycle and Apoptosis Analyses

The analyses were performed as described in the literature [58]. In particular, 1.5 × 10^5^ PANC-1 cells/well were seeded in 6-well plates and allowed to adhere overnight, followed by treatment with 3 µM and 6 µM of compound **12** for 48 h. For cell cycle analysis, cells were stained with propidium iodide (Sigma-Aldrich) and subjected to flow cytometry. Cell cycle phases were identified and quantified using the FlowJo X 10.0.7 software (Treestar, Ashland, OR, USA). For apoptosis analysis, cells were stained using the Annexin V-FITC Apoptosis Detection Kit I from BD Biosciences (Enzifarma, Porto, Portugal), according to the manufacturer’s instructions. The Accuri^TM^ C6 flow cytometer and the BD Accuri C6 software (BD Biosciences, Heidelberg, Germany) were used.

#### 3.2.4. Western Blot

PANC-1 (1.5 × 10^5^ cells/well) were seeded in six-well plates, allowed to adhere overnight, and treated with 3 µM and 6 µM of compound **12** for 48 h. Protein lysates were obtained in RIPA buffer with protease inhibitors (Sigma-Aldrich, Lisbon, Portugal) and quantified using Pierce^®^ bicinchoninic acid (BCA) protein assay reagents (Thermo Fisher Scientific, Porto Salvo, Portugal). Sodium dodecyl sulfate-polyacrylamide gel electrophoresis (SDS-PAGE) was performed, and proteins were transferred to a Whatman^®^ nitrocellulose membrane (Amersham Protran, GE Healthcare Life Sciences, Enzymatic, Portugal). Membranes were sectioned to allow the detection of p21 and cleaved PARP-1 proteins and blocked for 1 h with 5% skimmed milk. Membranes were then probed overnight with p21 (sc-6246) and cleaved PARP-1 (sc-56196) primary antibodies (from Santa Cruz Biotechnology, 1:100 dilution) and then with anti-mouse horseradish peroxidase (HRP)-conjugated secondary antibodies (from Abcam, Cambridge, UK, ab6789, 1:5000 dilution) for 2 h. Glyceraldehyde 3-phosphate dehydrogenase (GAPDH, from Santa Cruz Biotechnology, Heidelberg, Germany, sc-32233, 1:5000 dilution) antibody was used as loading control. The signal was detected with enhanced chemiluminescence (ECL) (Amersham, GE Healthcare Life Sciences, Enzymatic, Portugal) using the ChemiDoc^TM^ MP Imaging system (BioRad Laboratories, Amadora, Portugal).

#### 3.2.5. Wound Healing Assay

Totals of 6 × 10^5^ PANC-1 cells/well were grown to confluence in 6-well plates, and a fixed-width wound was created in the cell monolayer using a sterile 10 µL micropipette tip. Cells were treated with DMSO or 1.35 µM of compound **12** and images of the wound were captured at different time points (0, 6, and 24 h) using an inverted NIKON TE 2000-U microscope from Nikon Instruments Inc. (Izasa, Carnaxide, Portugal) at 100× magnification with a DXM1200F digital camera (Nikon Instruments Inc., Amstelveen, The Netherlands) and NIS-Elements microscope imaging software (version 4; Nikon Instruments Inc.). Wound closure was calculated by subtracting the wound area (measured using Fiji Software, version 1.54f) at the indicated time point of treatment from the wound area at the starting point (0 h).

#### 3.2.6. Combination Therapy with 5-Fluorouracil

PANC-1 (5.0 × 10^3^ cells/well) were seeded overnight in 96-well plates and then treated with DMSO (control), 1.35 µM of compound **12**, and/or increasing concentrations of 5-FU (3.75 to 60 µM), for 48 h. The effect on cell proliferation was analyzed by SRB assay. Mutually nonexclusive combination index (CI) and dose reduction index (DRI) of 5-FU were determined using CompuSyn software (version 1.0, ComboSyn, Inc., Paramus, NJ, USA), as described in [59,60]. Synergistic interactions of drugs were indicated as CI < 1, antagonist interactions as CI > 1.1, and additive effects as 1 < CI < 1.1.

#### 3.2.7. Statistical Analysis

Data were statistically analyzed using the GraphPad Prism (La Jolla, CA, USA; version 7.0) software. For comparison of multiple groups, statistical analysis relative to controls was performed using one-way or two-way ANOVA followed by post hoc Sidak’s or Dunnett’s multiple comparison tests. Statistical significance was set as * *p* < 0.05.

## 4. Conclusions

In the current study, a series of 22 new 1,2,3-triazole-substituted andrographolide derivatives was successfully prepared and screened for anticancer activity in several cancer cell lines. Most of the derivatives showed improved antiproliferative activities against several cancer cell lines compared to andrographolide (**1**). Compound **12**, bearing a (12*R*)-4-bromophenyl-1,2,3-triazole moiety, was selected for further studies, which revealed that **12** led to cell cycle arrest at the G2/M phase and can induce apoptosis in PANC-1 cells. Moreover, a synergistic inhibitory effect on the proliferation of PANC-1 cells was observed between compound **12** and 5-FU. These findings led us to conclude that compound **12** may be a valuable candidate for further studies aimed at the development of effective anticancer drugs.

## Figures and Tables

**Table 1:** Antiproliferative effect of compounds **1**–**26** on cancer (PANC-1, HCT116, A375, and MCF-7) and non-tumorigenic (CCD-18Co) cell lines.

Compound	Cell Line/IC_50_ (µM) ± SEM				
	PANC-1	HCT116	A375	MCF-7	CCD-18Co
**1**	3.1 ± 0.1	5.1 ± 1.5	4.0 ± 0.1	13.5 ± 0.5	6.2 ± 1.1
**2**	1.9 ± 0.2	1.4 ± 0.1	1.2 ± 0.2	4.9 ± 0.1	5.6 ± 1.3
**3**	1.9 ± 0.1	7.9 ± 2.1	1.3 ± 0.1	6.6 ± 0.1	3.8 ± 0.7
**4**	3.0 ± 0.2	1.5 ± 0.2	1.2 ± 0.2	6.9 ± 0.8	2.2 ± 0.8
**5**	8.5 ± 1.0	8.4 ± 1.1	7.7 ± 1.3	25.0 ± 3.0	7.1 ± 2.6
**6**	3.4 ± 0.9	1.2 ± 0.1	1.3 ± 0.3	3.8 ± 0.2	4.4 ± 0.2
**7**	4.1 ± 0.3	1.6 ± 0.4	3.6 ± 0.9	18.0 ± 0.1	12.9 ± 2.2
**8**	3.1 ± 0.4	2.1 ± 0.6	23.0 ± 2.0	15.5 ± 0.5	8.7 ± 1.9
**9**	2.0 ± 0.1	1.9 ± 0.6	2.1 ± 0.3	8.1 ± 0.1	3.8 ± 0.9
**10**	3.8 ± 0.7	2.2 ± 0.1	7.7 ± 1.0	7.8 ± 0.8	7.2 ± 0.6
**11**	3.4 ± 0.6	1.9 ± 0.3	8.2 ± 0.1	4.2 ± 0.8	3.3 ± 0.4
**12**	3.4 ± 0.9	1.7 ± 0.2	1.7 ± 0.0	6.8 ± 0.3	9.6 ± 2.3
**13**	6.5 ± 0.4	4.8 ± 1.3	6.8 ± 0.9	26.0 ± 3.0	30.0 ± 2.0
**14**	2.5 ± 0.3	2.0 ± 0.6	1.7 ± 0.2	7.9 ± 0.6	3.7 ± 0.9
**15**	3.3 ± 0.9	1.3 ± 0.1	1.2 ± 0.2	5.6 ± 0.2	6.2 ± 1.2
**16**	2.3 ± 0.2	2.3 ± 0.9	1.5 ± 0.2	9.3 ± 0.1	6.8 ± 1.0
**17**	2.1 ± 0.1	1.7 ± 0.5	0.9 ± 0.1	5.6 ± 0.2	4.4 ± 0.9
**18**	3.5 ± 1.6	3.7 ± 0.5	6.6 ± 0.8	9.5 ± 0.5	5.1 ± 0.1
**19**	3.1 ± 0.8	4.1 ± 0.2	7.1 ± 0.1	7.9 ± 1.5	6.6 ± 0.9
**20**	3.0 ± 1.1	1.4 ± 0.3	3.3 ± 1.3	28.0 ± 2.0	3.5 ± 0.3
**21**	3.5 ± 0.3	1.2 ± 0.2	1.3 ± 0.3	10.4 ± 0.7	4.4 ± 0.8
**22**	5.0 ± 0.4	1.6 ± 0.3	1.3 ± 0.2	5.9 ± 0.1	3.2 ± 0.9
**23**	6.3 ± 1.9	2.8 ± 0.8	3.3 ± 1.0	11.0 ± 0.2	4.1 ± 0.9
**24**	1.8 ± 0.1	6.7 ± 3.2	1.3 ± 0.3	7.0 ± 0.2	4.6 ± 1.4
**25**	4.5 ± 2.0	1.7 ± 0.3	1.8 ± 0.3	6.9 ± 0.1	8.9 ± 0.2
**26**	4.3 ± 1.4	1.3 ± 0.1	1.4 ± 0.1	6.1 ± 0.2	6.2 ± 1.4
Etoposide	2.8 ± 0.05	0.65 ± 0.09	0.82 ± 0.07	1.8 ± 0.03	0.76 ± 0.01

PANC-1, pancreatic ductal adenocarcinoma cells; HCT116, colorectal adenocarcinoma cells; A375, melanoma cells; MCF-7, breast adenocarcinoma cells; CCD-18Co, non-tumorigenic colon cells; IC_50_ values are presented as the mean ± SEM of four to six independent experiments.

## Data Availability

Data is contained in the paper.

## Supplementary Materials

The following supporting information can be downloaded at: https://www.mdpi.com/article/10.3390/ph18050750/s1. Figures S1–S25: ^1^H and ^13^C NMR spectra of selected compounds; Figure S26: Uncropped blots showing cleaved PARP-1, p21 and GAPDH.
