# Supplementary material for: Exploring the Anticancer Properties of 1,2,3-Triazole-Substituted Andrographolide Derivatives
Source: Pharmaceuticals (Basel). 2025 May 19;18(5):750. doi: 10.3390/ph18050750 (PMC12114840; doi:10.3390/ph18050750)
Supplement: Supplementary file 1 [file pharmaceuticals-18-00750-s001.zip › pharmaceuticals-3620457-supplementary.pdf]

Electronic Supplementary Information

Exploring the Anticancer Properties of 1,2,3-Triazole-Substituted Andrographolide Derivatives

Joana Ribeiro[a], Juliana Calheiros[b], Rita Silva[b], Bruno M. F. Goncalves[a], Carlos A. M. Afonso[a,*]
Lucília Saraiva[b,*] and Maria-José U. Ferreira[a,*]

[a] Research Institute for Medicines (iMed.ULisboa), Faculty of Pharmacy, Universidade de Lisboa, Av. Prof. Gama Pinto, 1649-003 Lisbon, Portugal
[b] LAQV/REQUIMTE, Laboratório de Microbiologia, Departamento de Ciências Biológicas, Faculdade de Farmácia, Universidade do Porto, 4050-313 Porto, Portugal

Table of contents

1. NMR data of parental compound 1 3

2. Representative ^{1}H and ^{13}C NMR spectra 5

3. Uncropped blots 18

4. References 18

1. NMR data of parental compound 1

White amorphous powder; ^{1}H-NMR (300 MHz, DMSO-d6) δ = 6.62 (1H, *td*, J = 6.8, 1.7 Hz, H-12), 5.70 (1H, *d*, J = 6.1 Hz, H-14-OH), 5.04 (1H, *d*, J = 4.9 Hz, H-3-OH), 4.91 (1H, *br t*, J = 6.1 Hz, H-14), 4.81 (1H, *br s*, H-17a), 4.63 (1H, *br s*, H-17b), 4.39 (1H, *dd*, J = 9.9, 6.1 Hz, H-15a), 4.12 (1H, *dd*, J = 7.5, 2.9 Hz, H-19-OH), 4.03 (1H, *dd*, J = 9.9, 2.1 Hz, H-15b), 3.84 (1H, *dd*, J = 11.0, 2.9 Hz, H-19a), 3.29-3.19 (2H, *m*, H-3 and H-19b), 2.46 (2H, *m*, H-11), 2.32 (2H, *m*, H-7), 1.95 (2H, *m*, H-2), 1.74 (2H, *m*, H-6), 1.63 (2H, *m*, H-1), 1.30 (1H, *m*, H-9), 1.20 (1H, *m*, H-5), 1.08 (3H, *s*, H-18), 0.66 (3H, *s*, H-20) ppm. ^{13}C-NMR (75 MHz, DMSO-d6) δ = 169.9 (C-16), 147.6 (C-12), 146.3 (C-8), 129.0 (C-13), 108.2 (C-17), 78.4 (C-3), 74.3 (C-15), 64.5 (C-14), 62.6 (C-19), 55.5 (C-9), 54.4 (C-5), 42.3 (C-4), 38.6 (C-10), 37.5 (C-7), 36.5 (C-1), 27.9 (C-2), 23.9 (C-6 and C-11), 23.0 (C-18), 14.7 (C-20) ppm. ESI-MS (positive mode) m/z (rel. Int) 351 $[M + H]^{+}$. These data are in agreement with the literature [1,2].

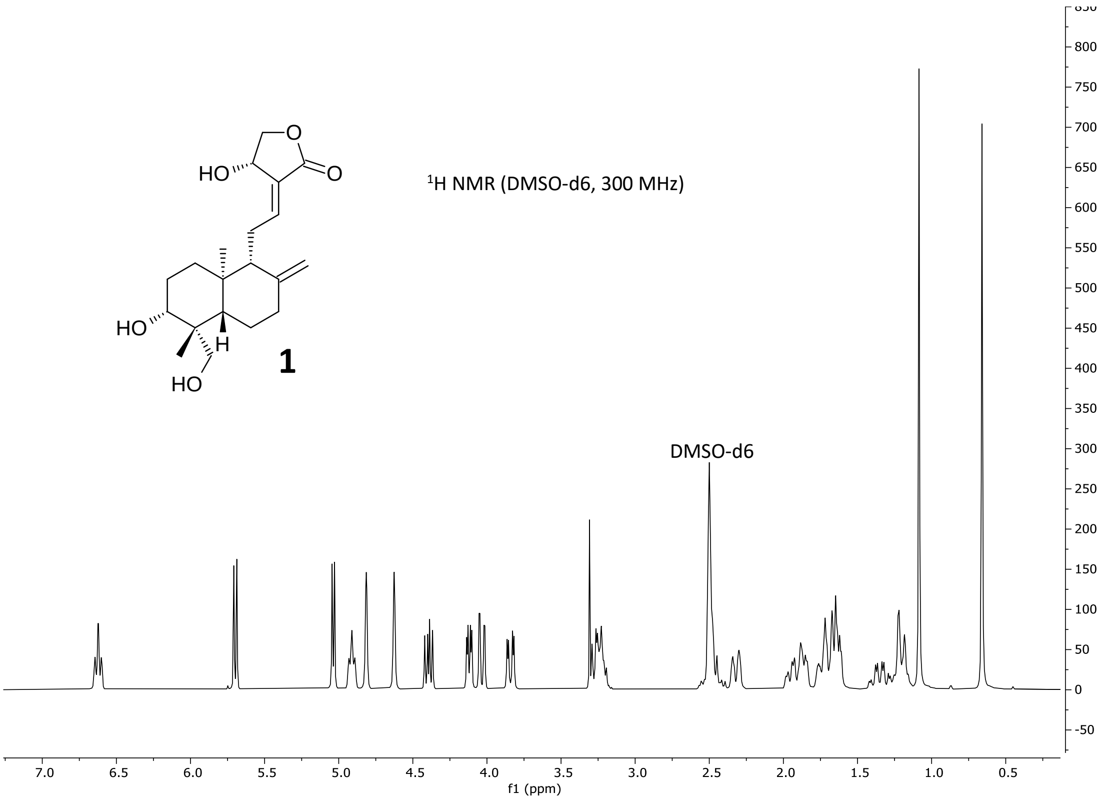

Figure S1: ^{1}H-NMR spectrum of compound **1** (300 MHz, DMSO-d6)

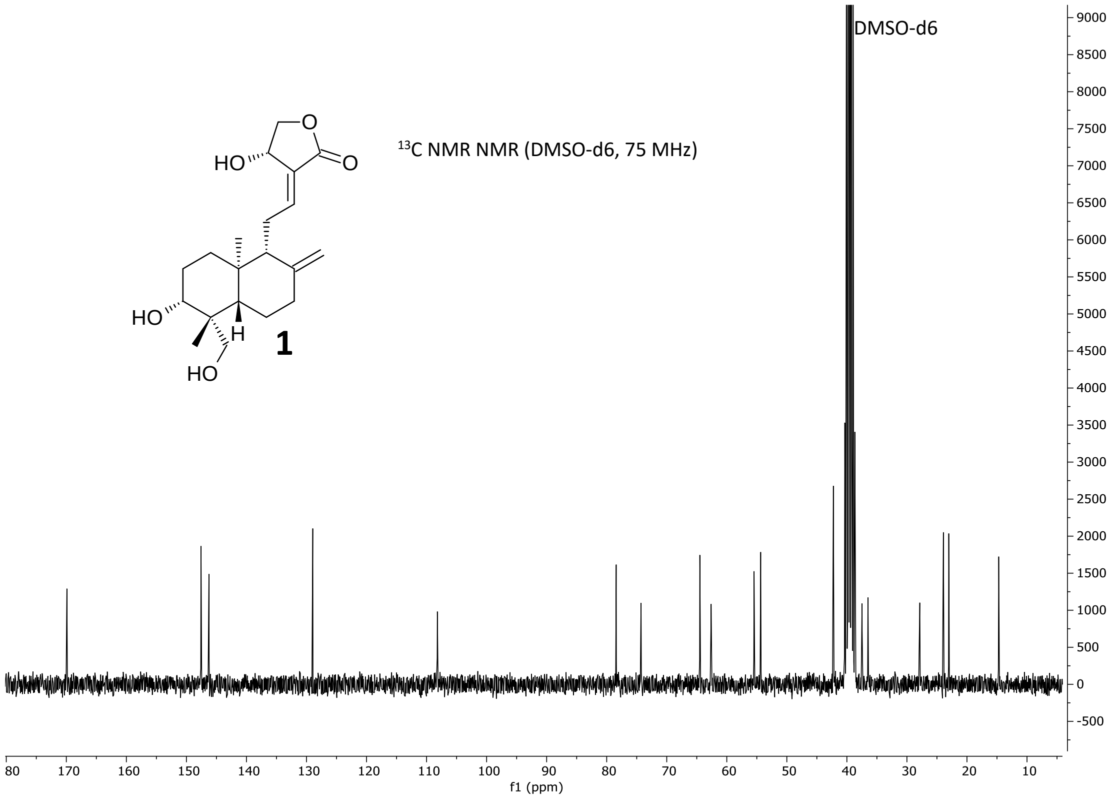

Figure S2: ^{13}C-NMR spectrum of compound **1** (75 MHz, DMSO-d6)

2. Representative ^{1}H and ^{13}C NMR spectra

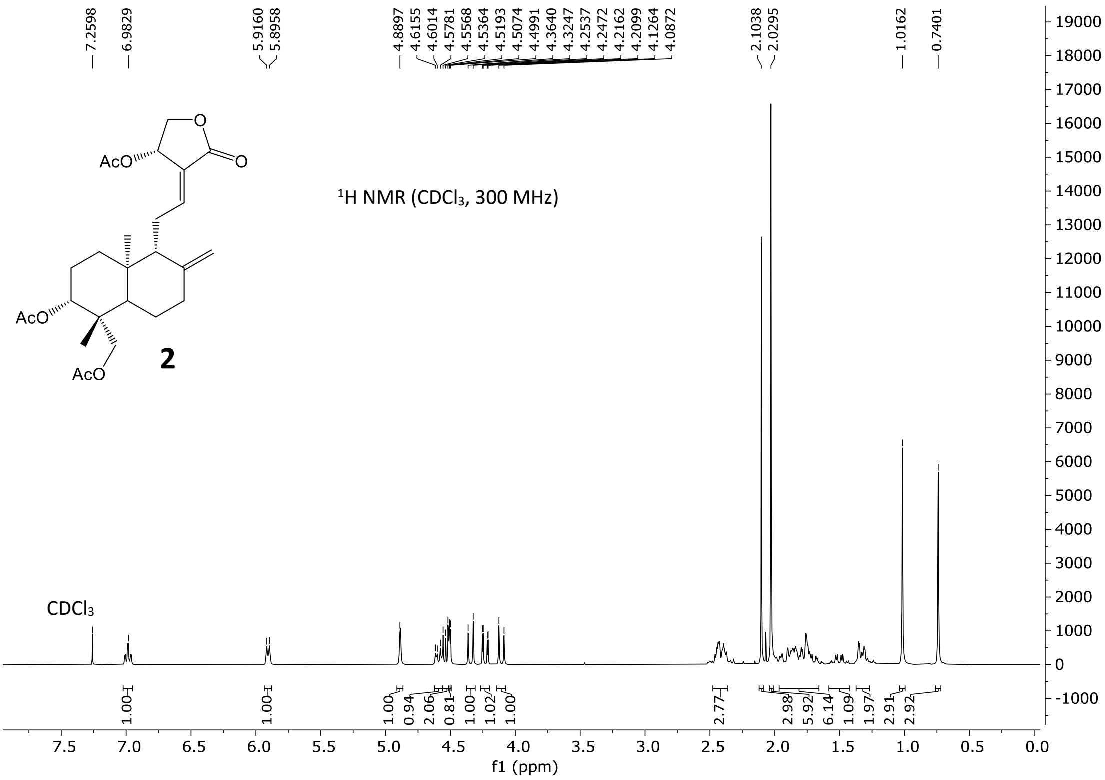

Figure S3: ^{1}H-NMR spectrum of compound **2** (300 MHz, $CDCl_3$)

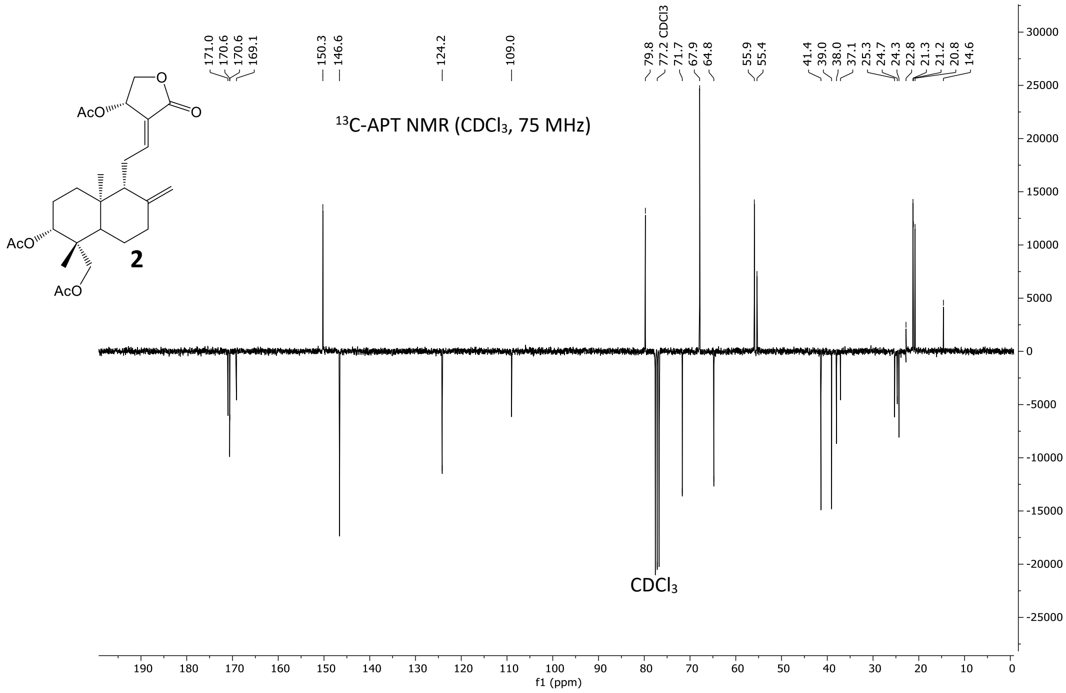

Figure S4: ^{13}C-APT NMR spectrum of compound **2** (75 MHz, $CDCl_3$). Quaternary (C) and methylene (CH_2) carbons are displayed as negative signals, while methine (CH) and methyl (CH_3) carbons are displayed as positive signals.

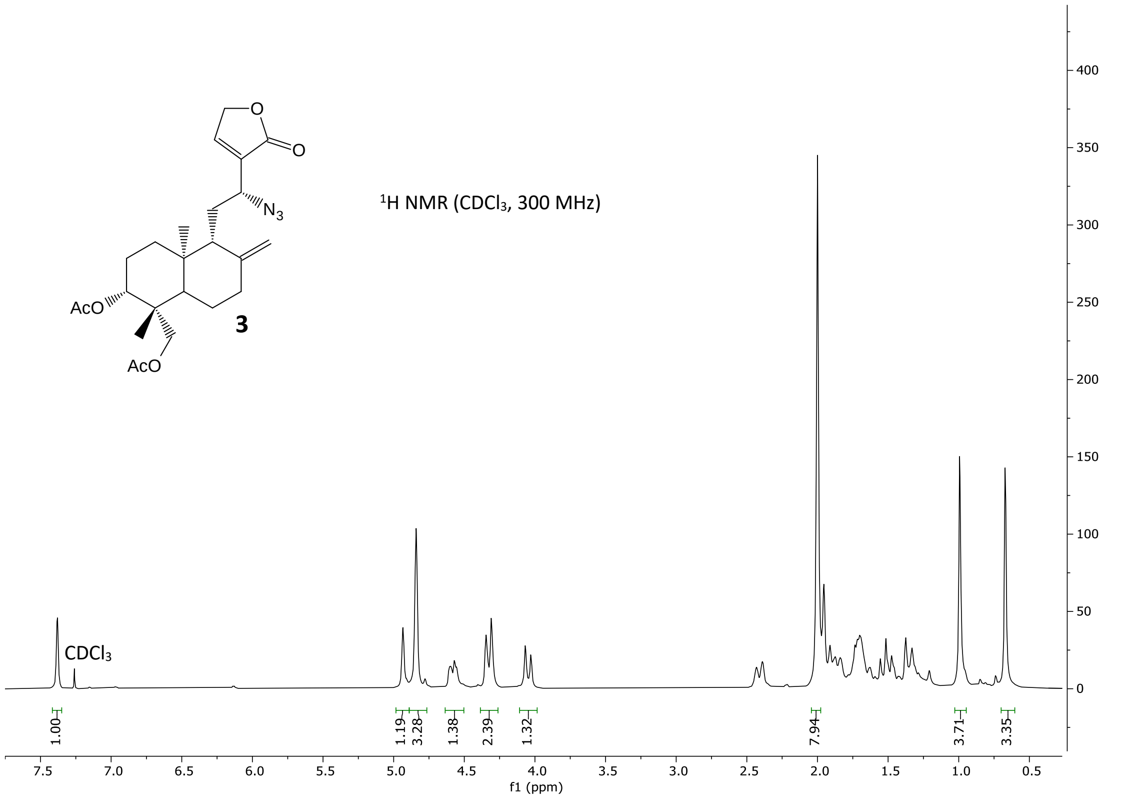

Figure S5: ^{1}H-NMR spectrum of compound **3** (300 MHz, $CDCl_3$)

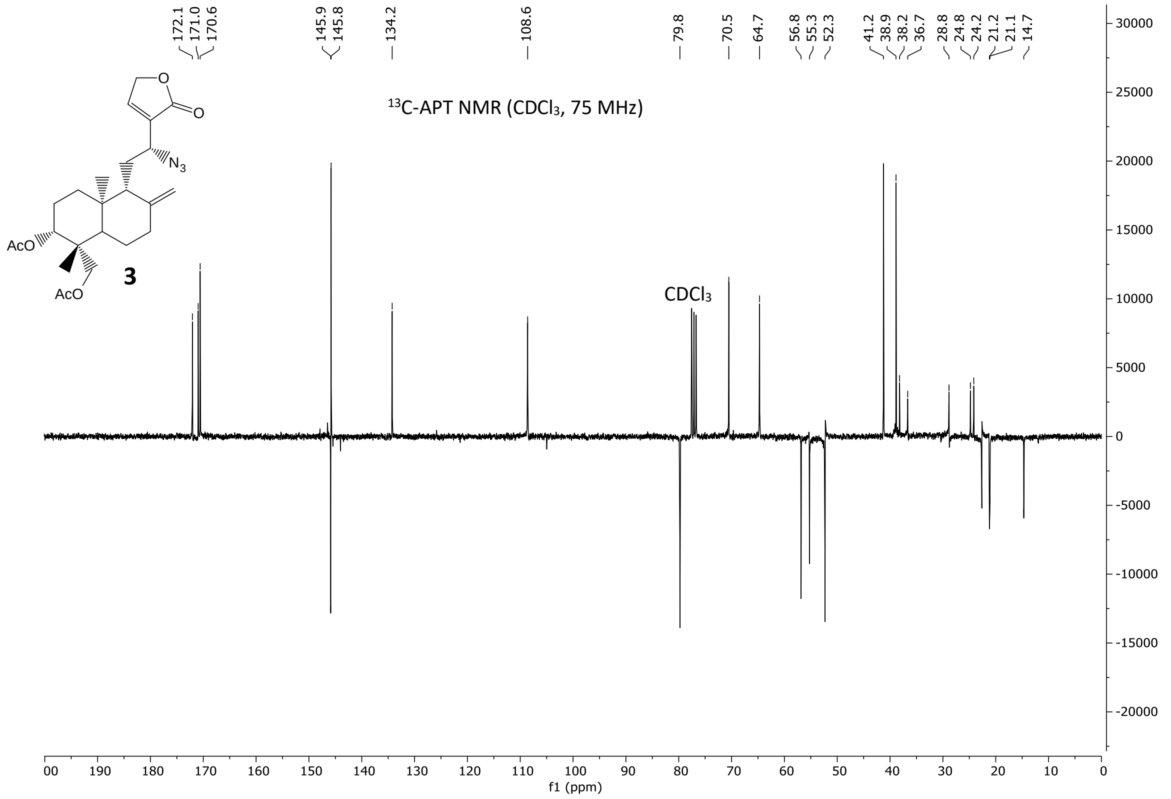

Figure S6: ^{13}C-APT NMR spectrum of compound **3** (75 MHz, $CDCl_3$). Quaternary (C) and methylene (CH_2) carbons are displayed as positive signals, while methine (CH) and methyl (CH_3) carbons are displayed as negative signals.

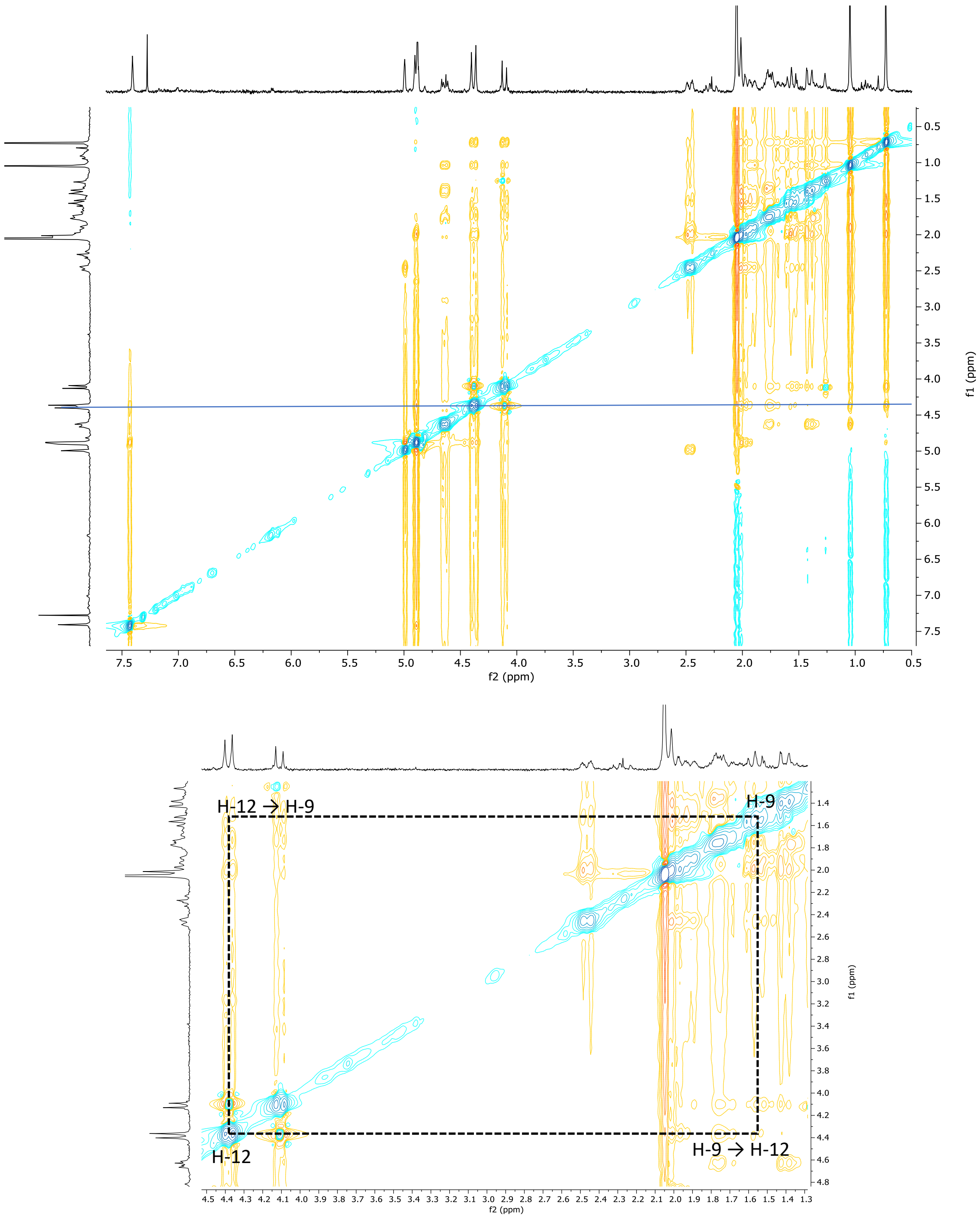

Figure S7: NOESY spectrum and expansion of compound **3** (300 MHz, $CDCl_3$)

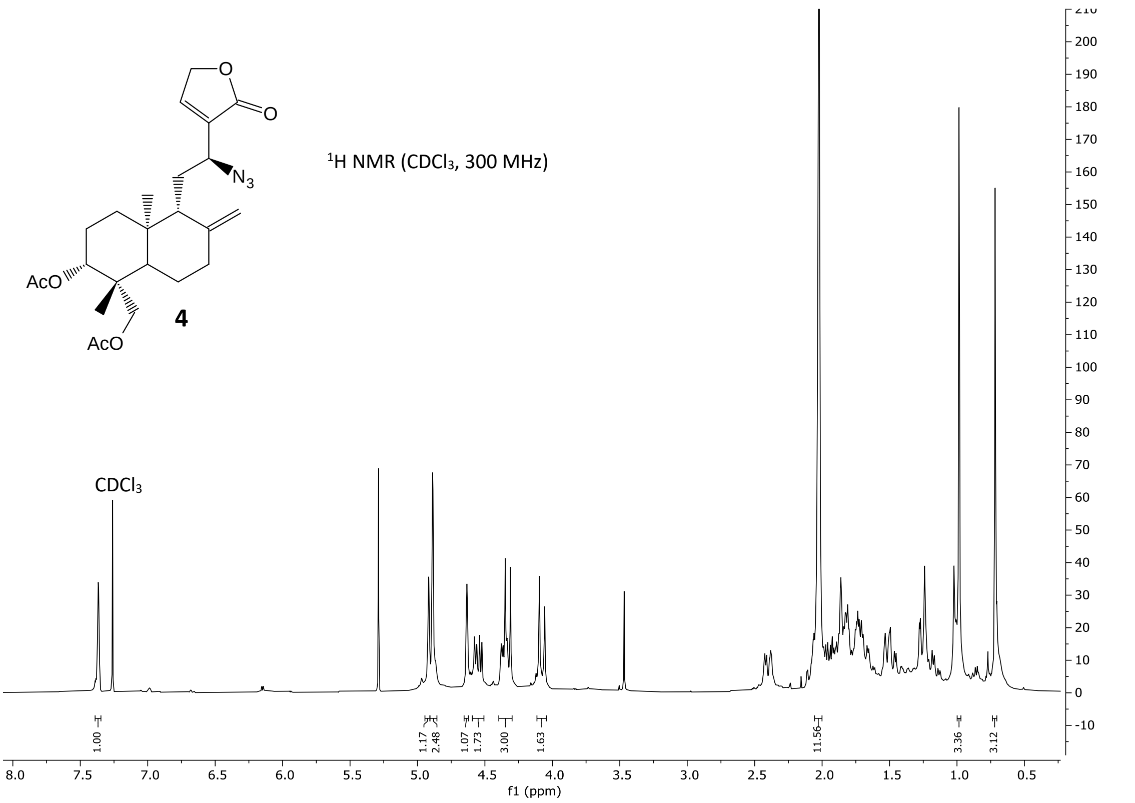

Figure S8: ^{1}H-NMR spectrum of compound **4** (300 MHz, $CDCl_3$)

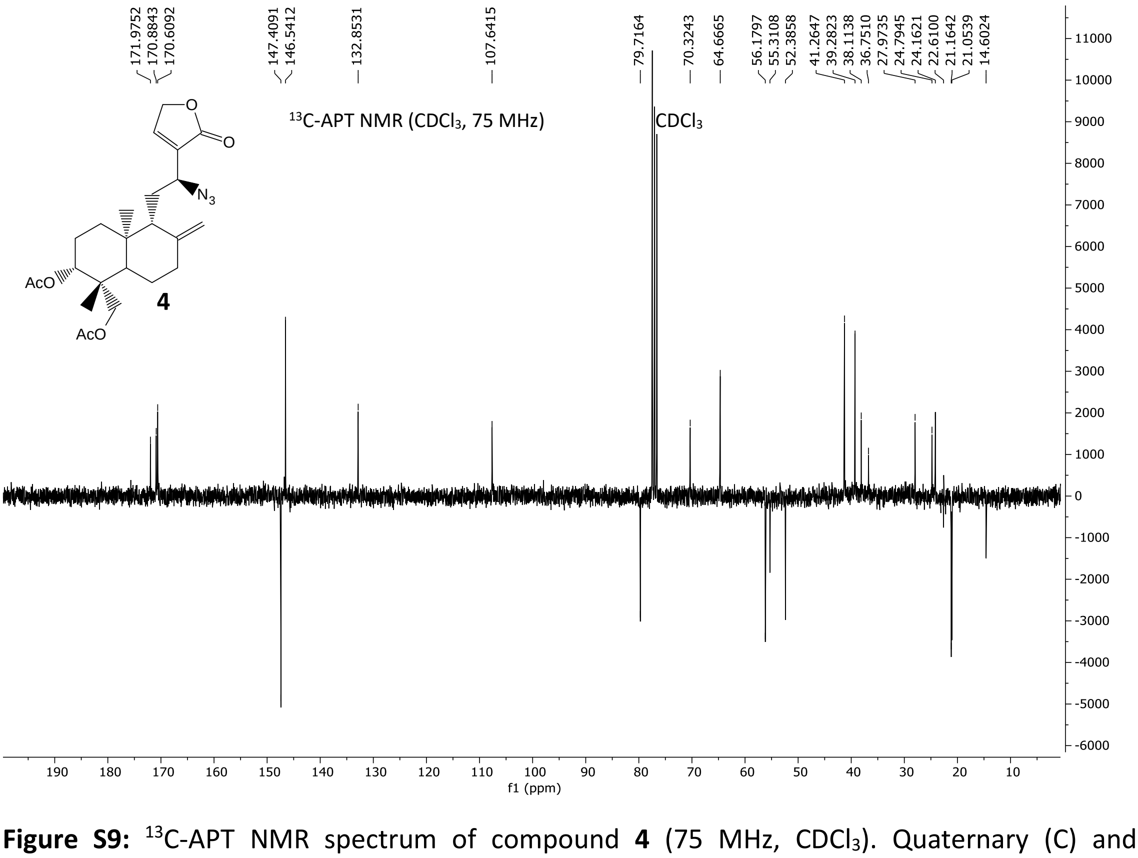

Figure S9: ^{13}C-APT NMR spectrum of compound **4** (75 MHz, $CDCl_3$). Quaternary (C) and methylene (CH_2) carbons are displayed as positive signals, while methine (CH) and methyl (CH_3) carbons are displayed as negative signals.

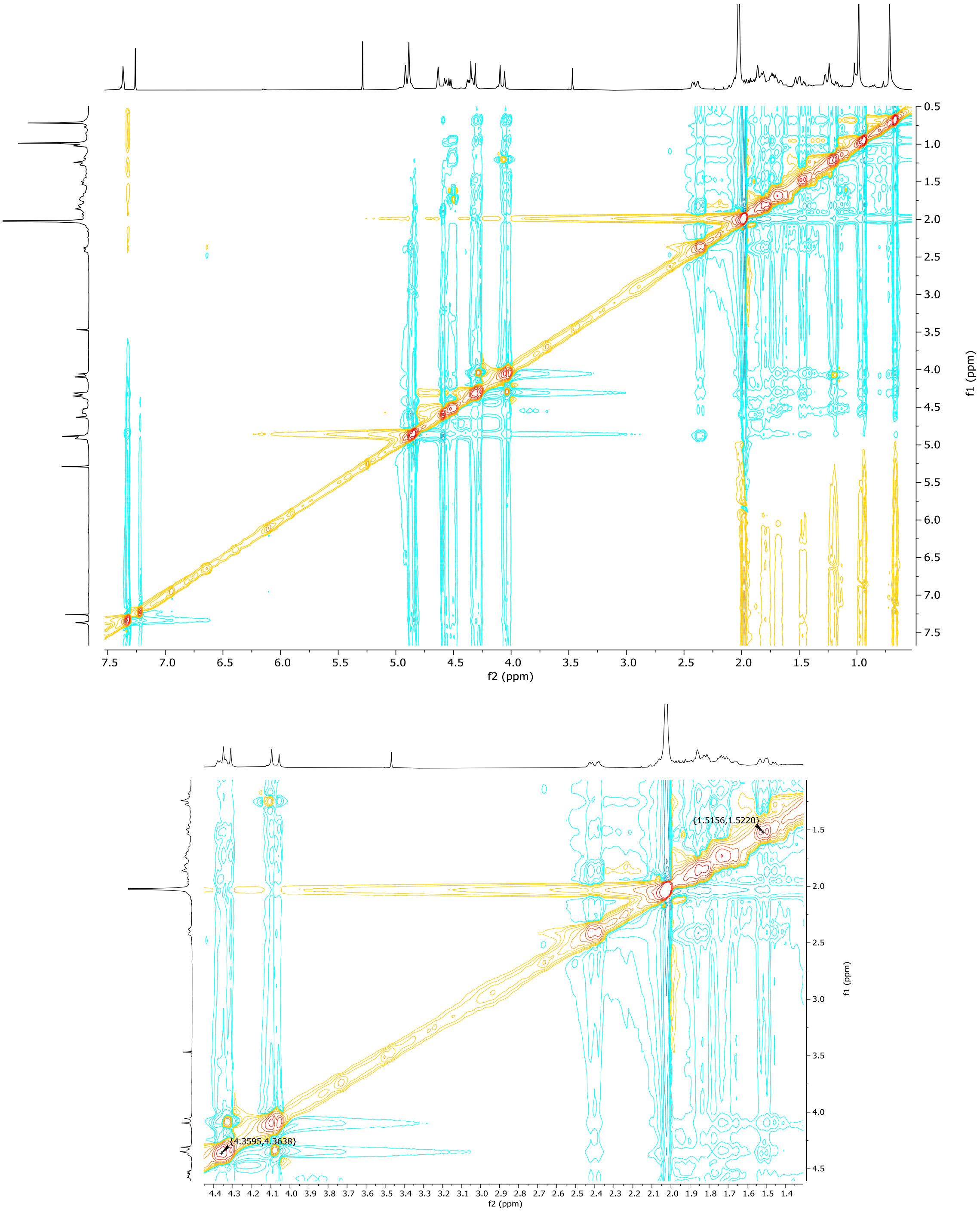

Figure S10: NOESY spectrum and expansion of compound **4** (300 MHz, $CDCl_3$)

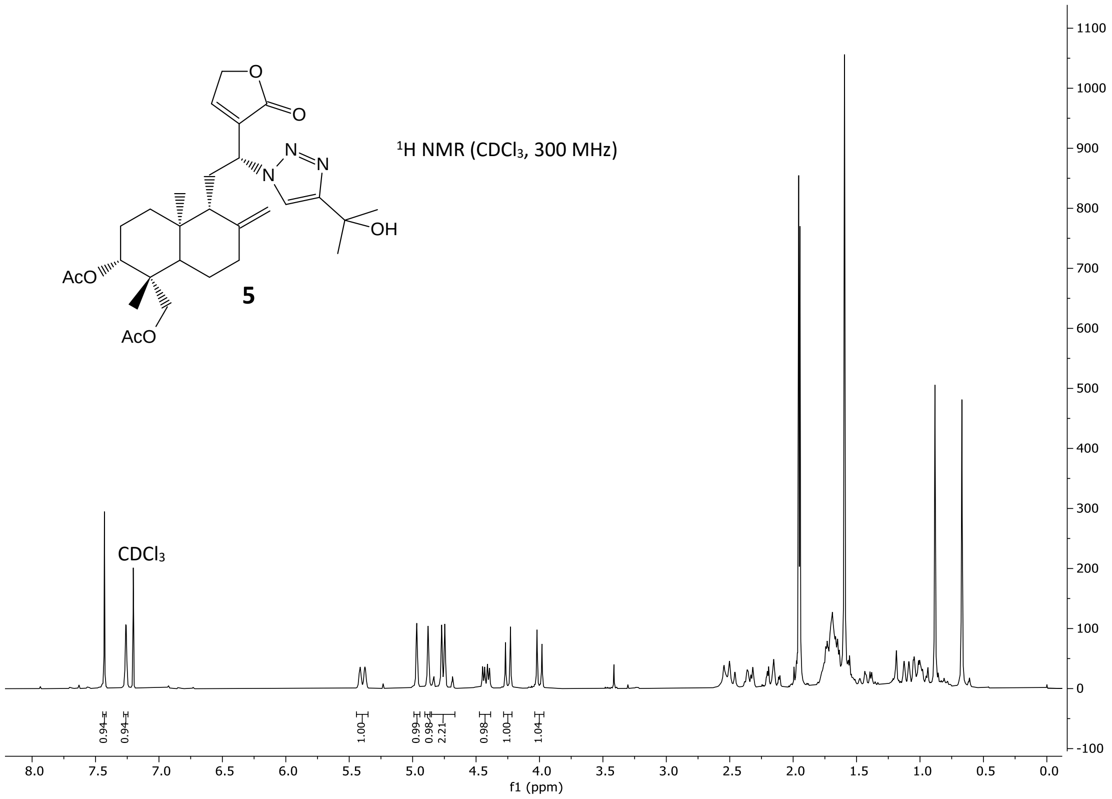

Figure S11: ^{1}H-NMR spectrum of compound **5** (300 MHz, $CDCl_3$)

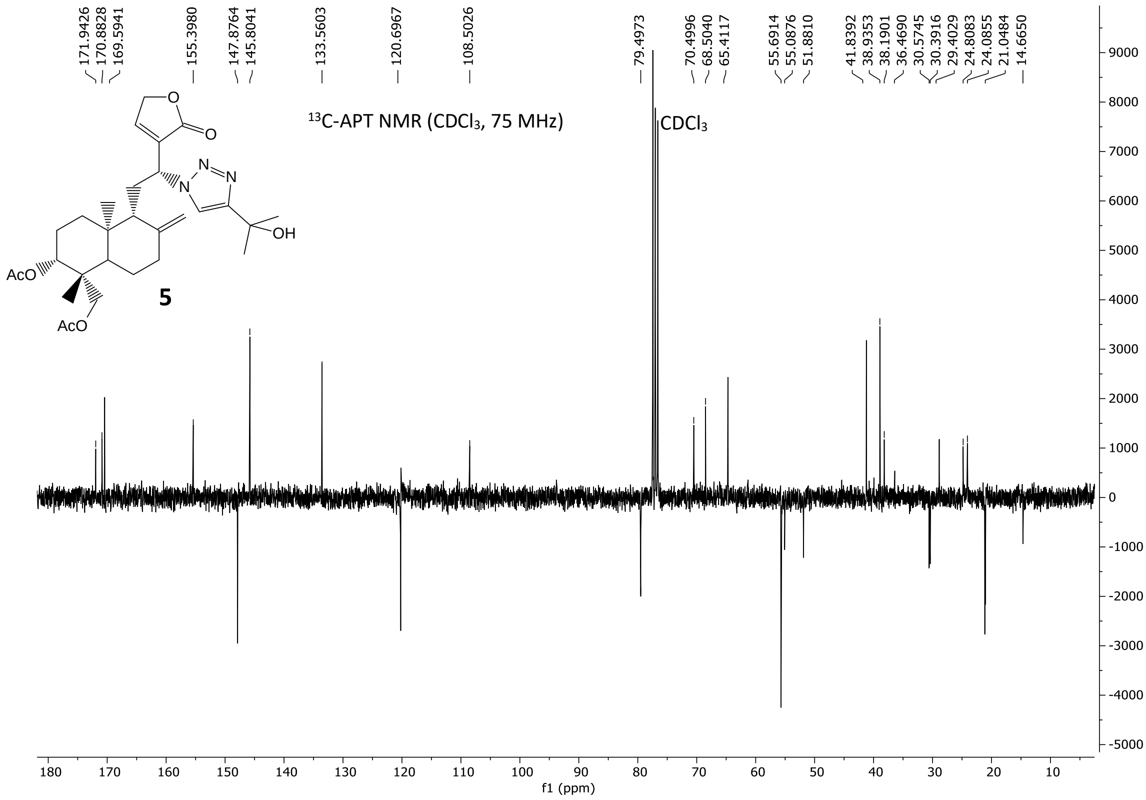

Figure S12: ^{13}C-APT NMR spectrum of compound **5** (75 MHz, $CDCl_3$) Quaternary (C) and methylene (CH_2) carbons are displayed as positive signals, while methine (CH) and methyl (CH_3) carbons are displayed as negative signals.

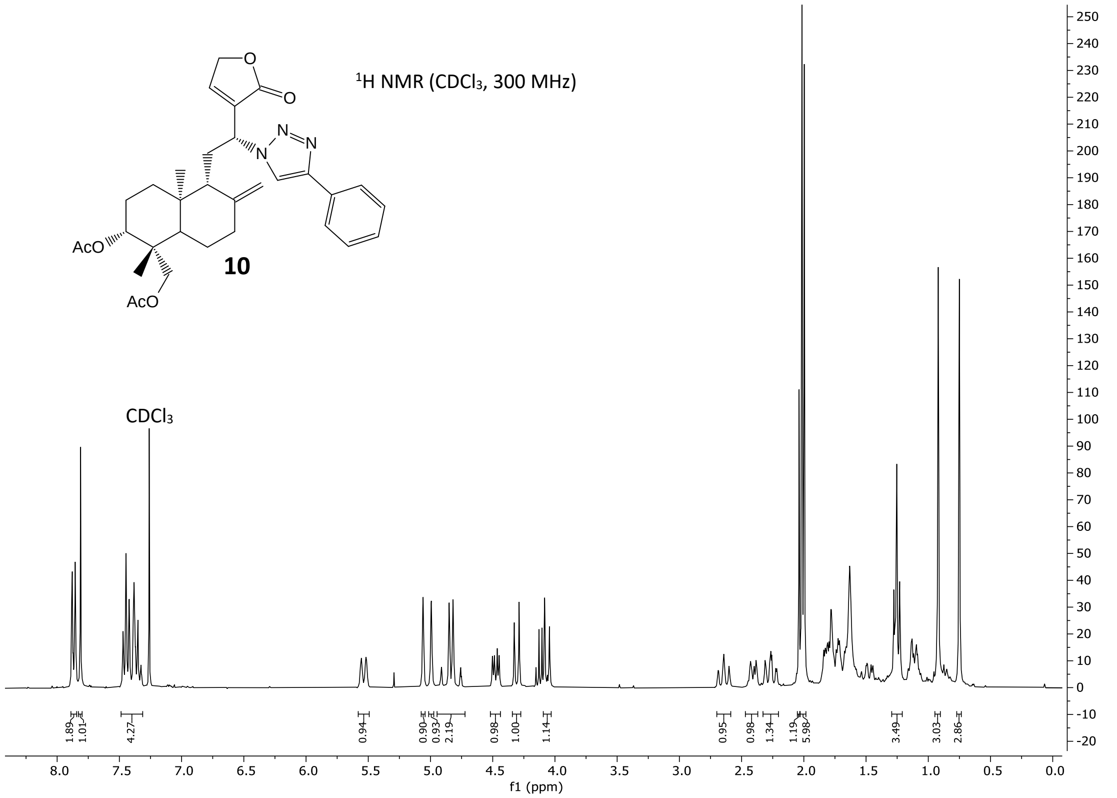

Figure S13: ^{1}H-NMR spectrum of compound **10** (300 MHz, $CDCl_3$)

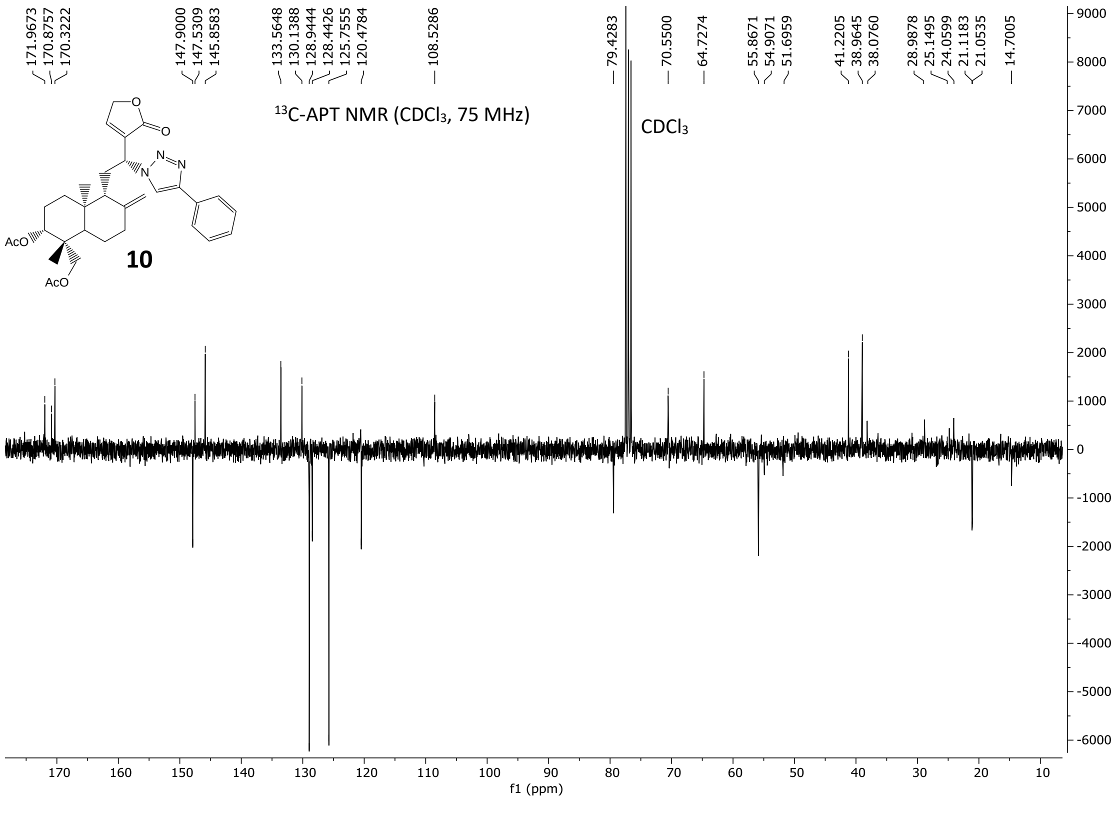

Figure S14: ^{13}C-APT NMR spectrum of compound **10** (75 MHz, $CDCl_3$). Quaternary (C) and methylene (CH_2) carbons are displayed as positive signals, while methine (CH) and methyl (CH_3) carbons are displayed as negative signals.

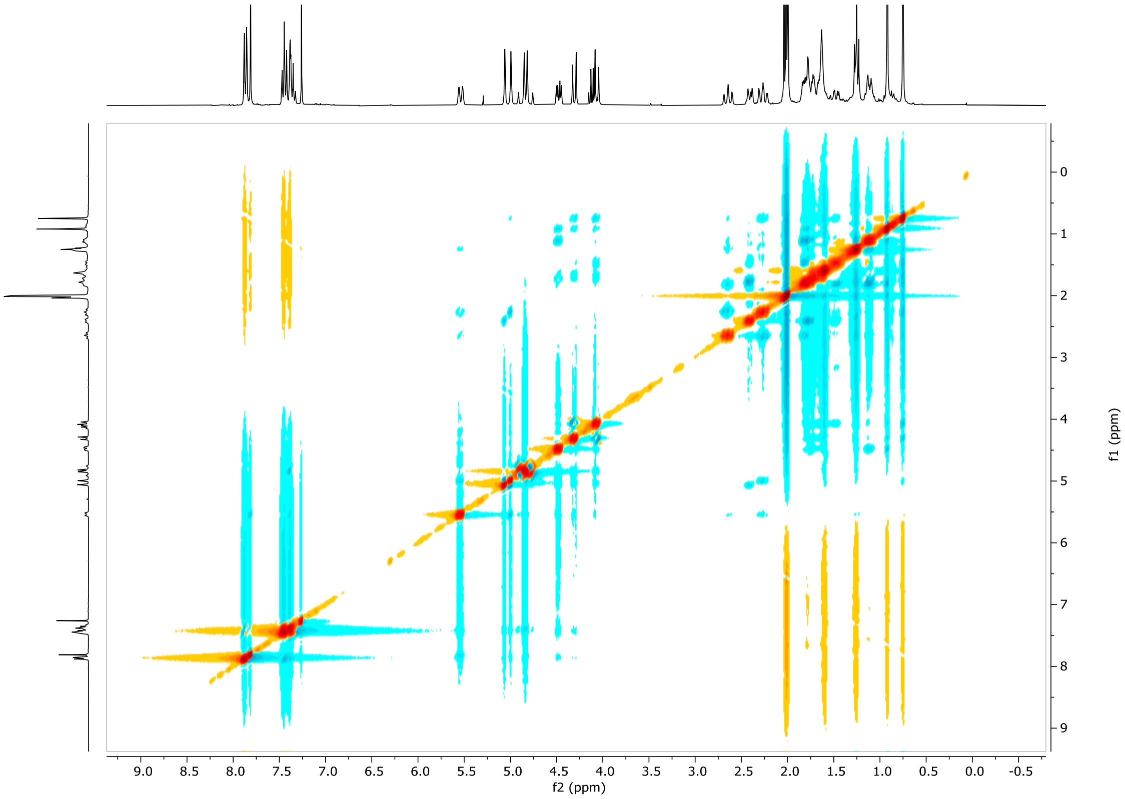

Figure S15: NOESY spectrum and expansion of compound **10** (300 MHz, $CDCl_3$)

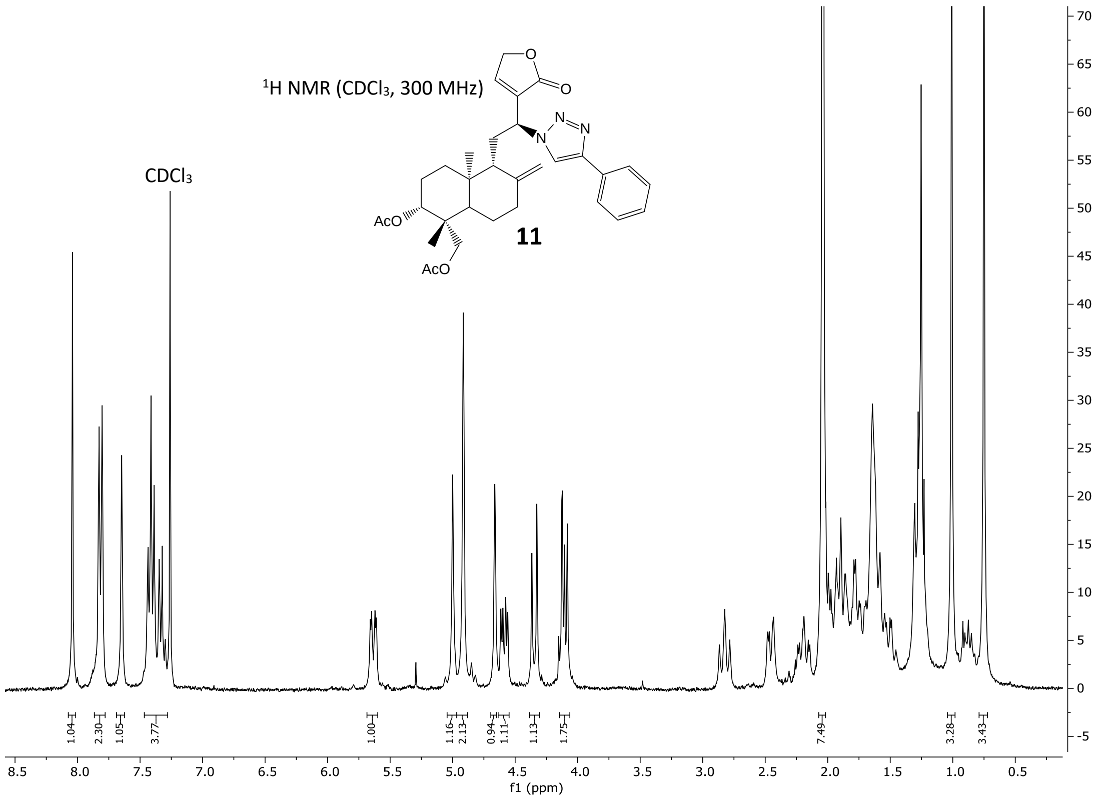

Figure S16: ^{1}H-NMR spectrum of compound **11** (300 MHz, $CDCl_3$)

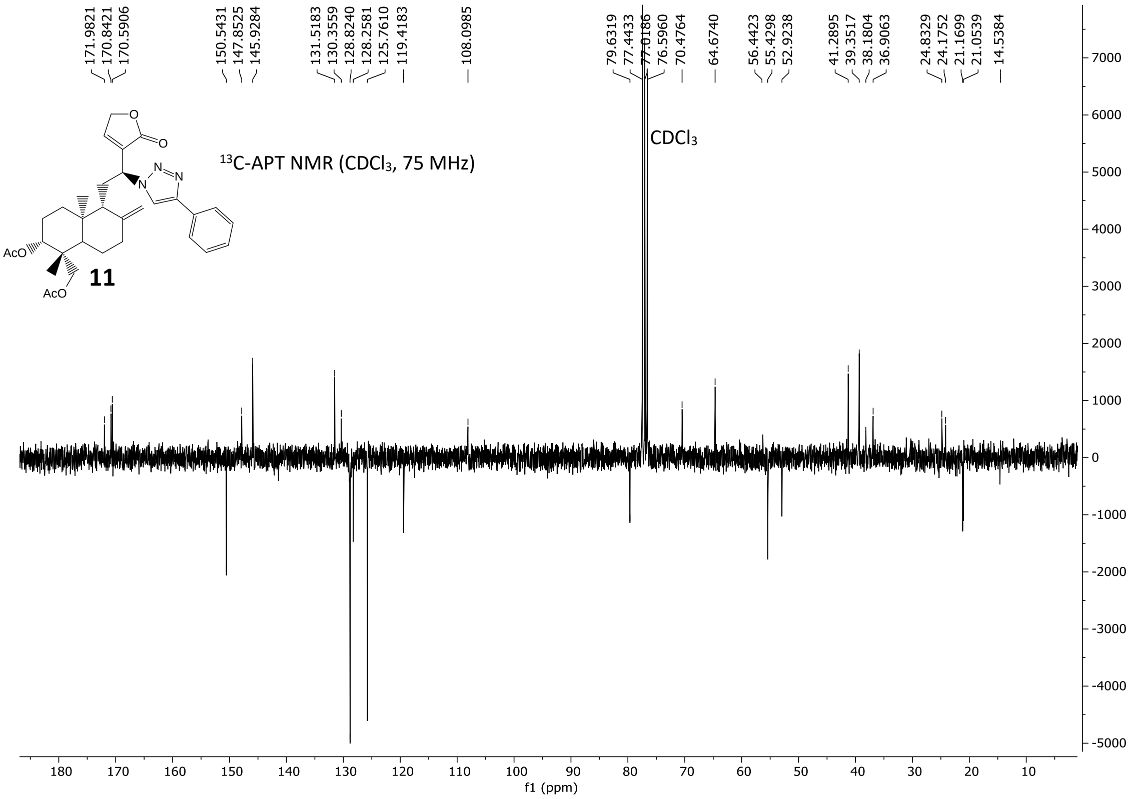

Figure S17: ^{13}C-APT NMR spectrum of compound **11** (75 MHz, $CDCl_3$). Quaternary (C) and methylene (CH_2) carbons are displayed as positive signals, while methine (CH) and methyl (CH_3) carbons are displayed as negative signals.

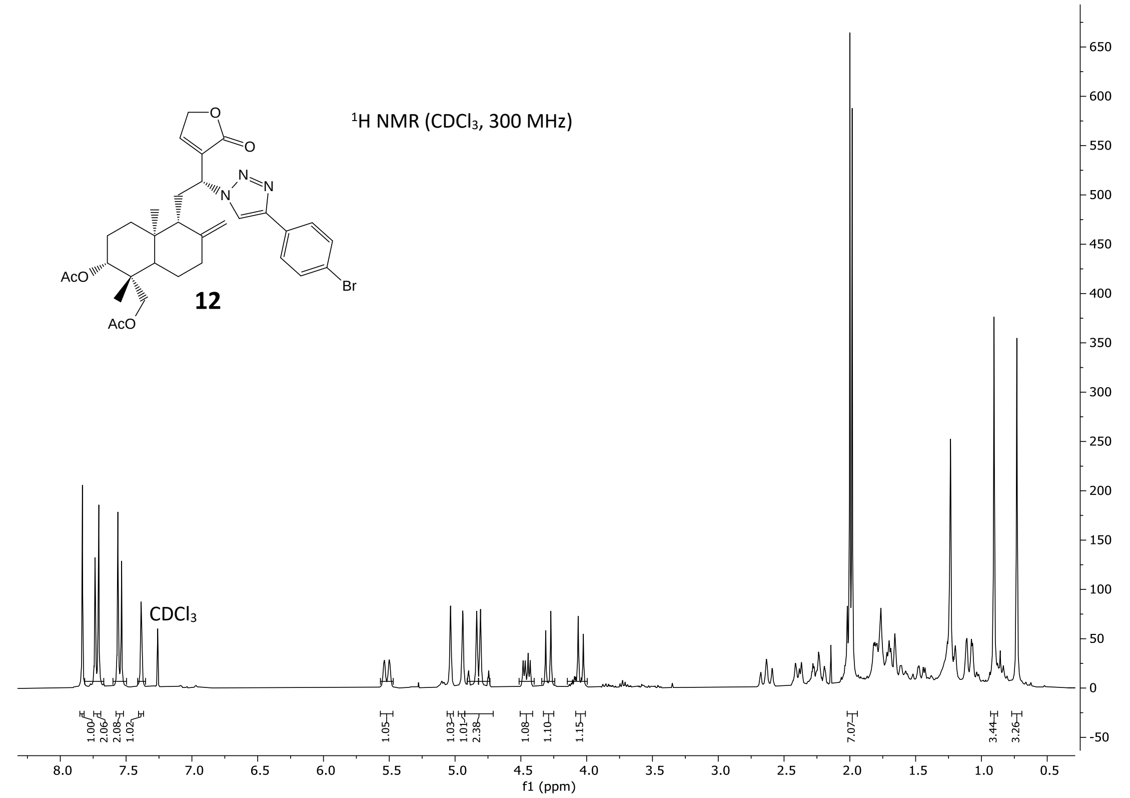

Figure S18: ^{1}H-NMR spectrum of compound **12** (300 MHz, $CDCl_3$)

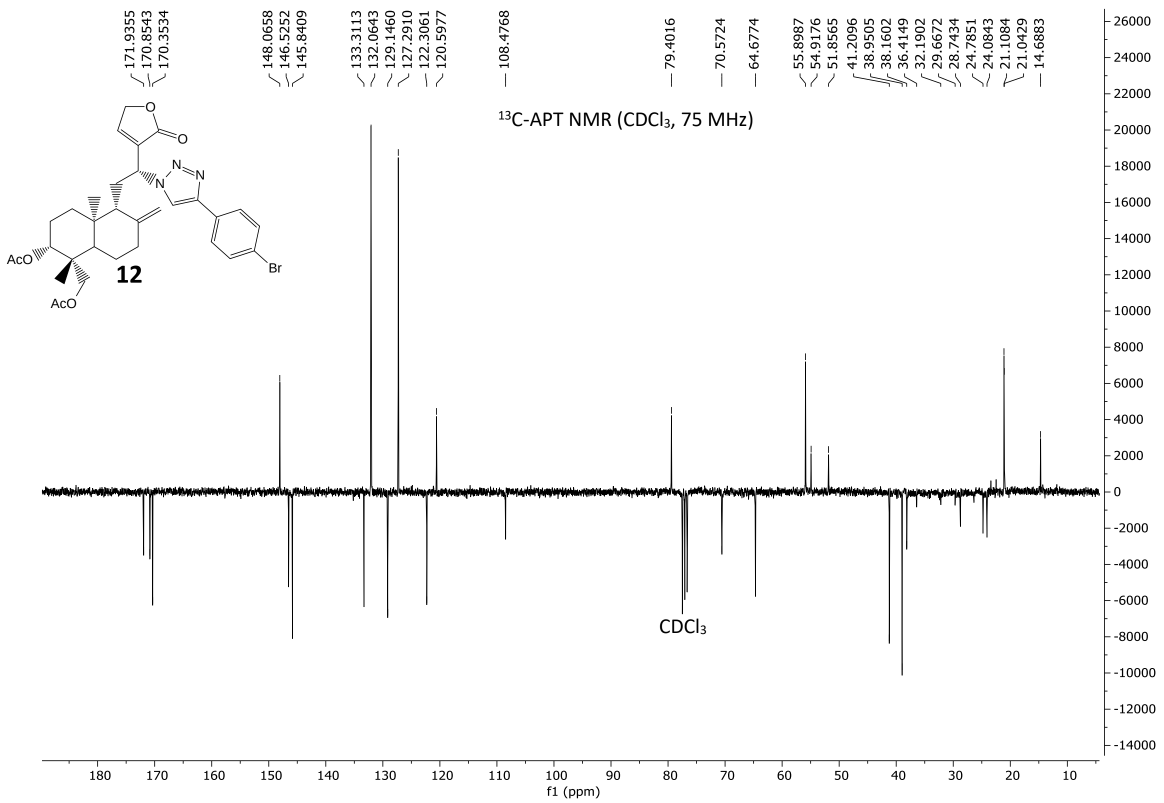

Figure S19: ^{13}C-APT NMR spectrum of compound **12** (75 MHz, $CDCl_3$). Quaternary (C) and methylene (CH_2) carbons are displayed as negative signals, while methine (CH) and methyl (CH_3) carbons are displayed as positive signals.

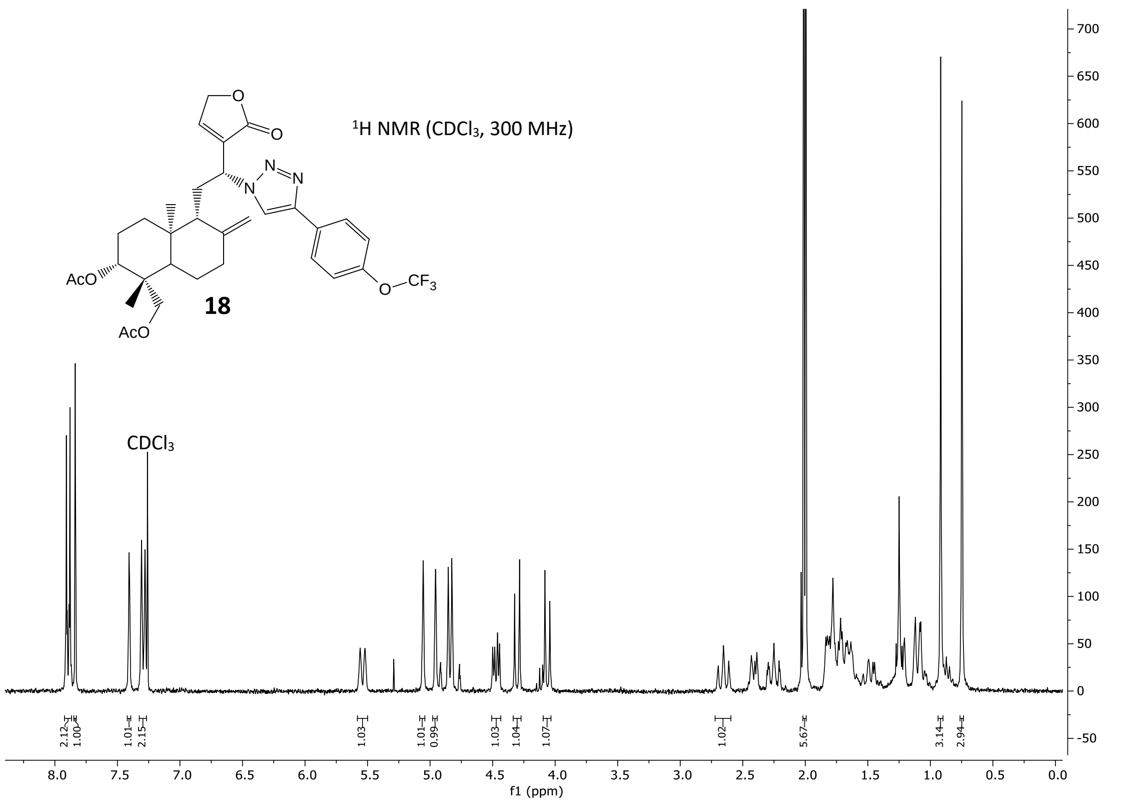

Figure S20: ^{1}H-NMR spectrum of compound **18** (300 MHz, $CDCl_3$)

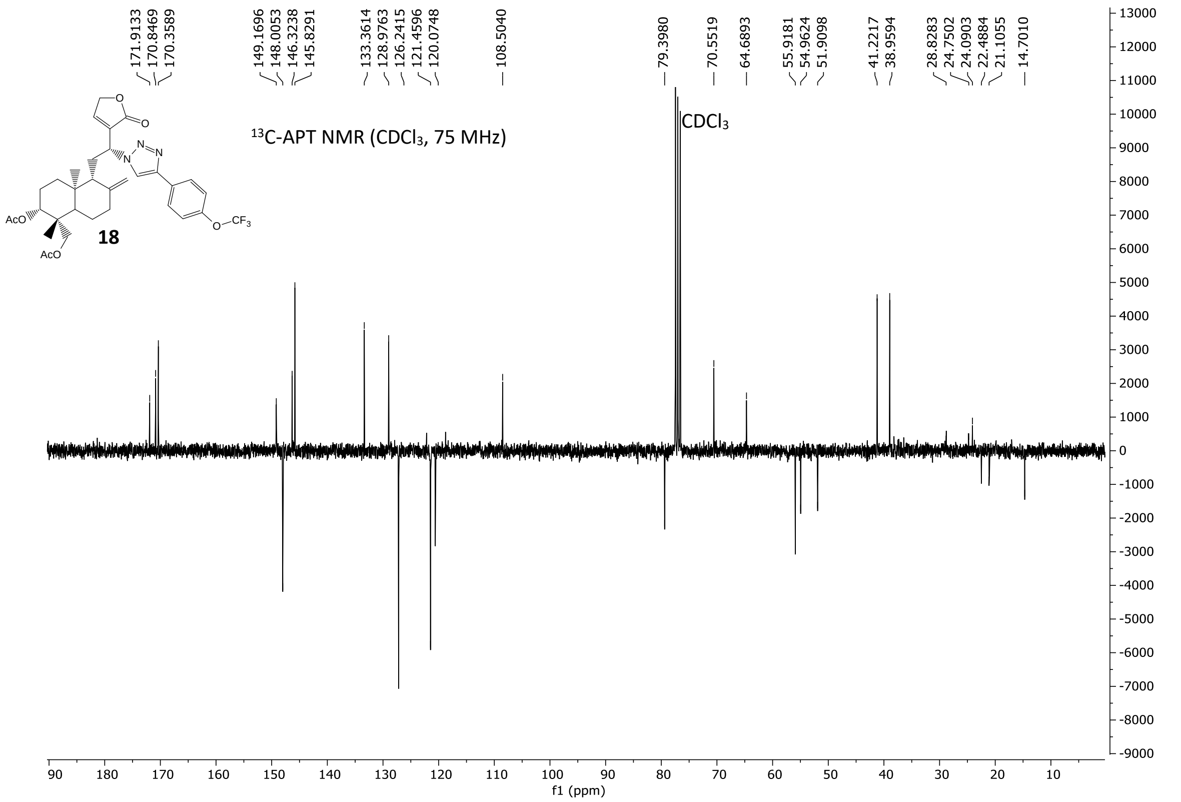

Figure S21: ^{13}C-APT NMR spectrum of compound **18** (75 MHz, $CDCl_3$). Quaternary (C) and methylene (CH_2) carbons are displayed as positive signals, while methine (CH) and methyl (CH_3) carbons are displayed as negative signals.

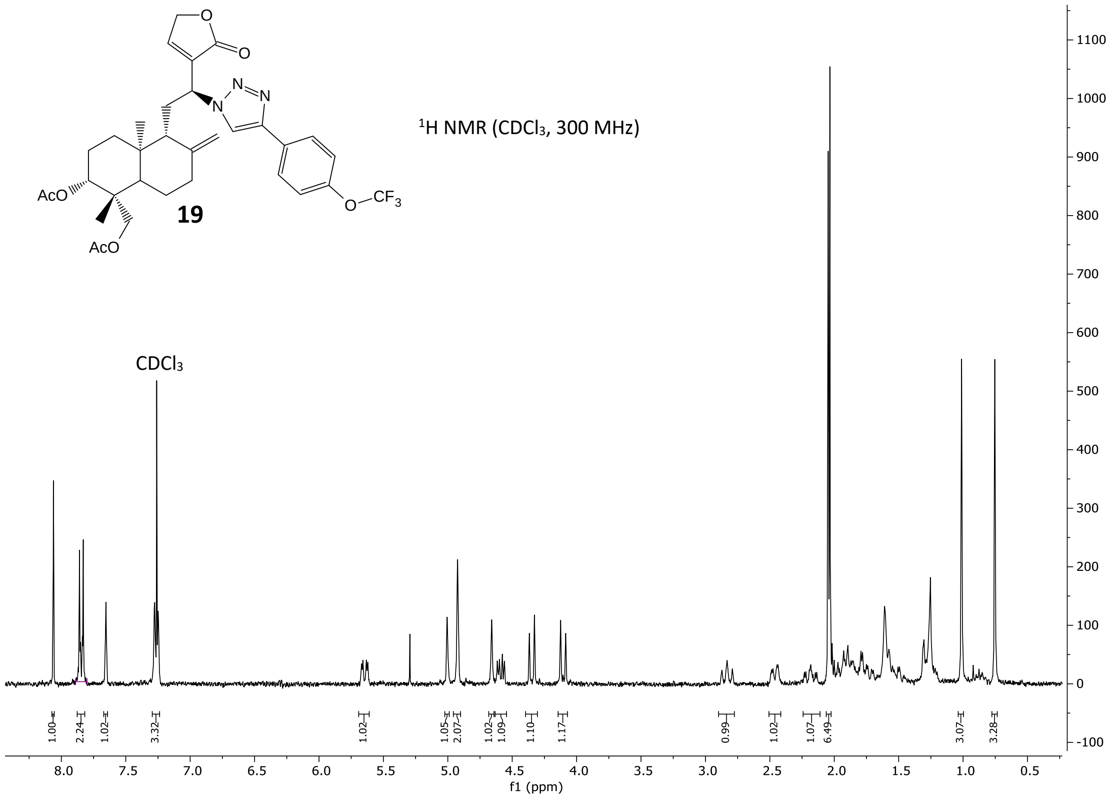

Figure S22: ^{1}H-NMR spectrum of compound **19** (300 MHz, $CDCl_3$)

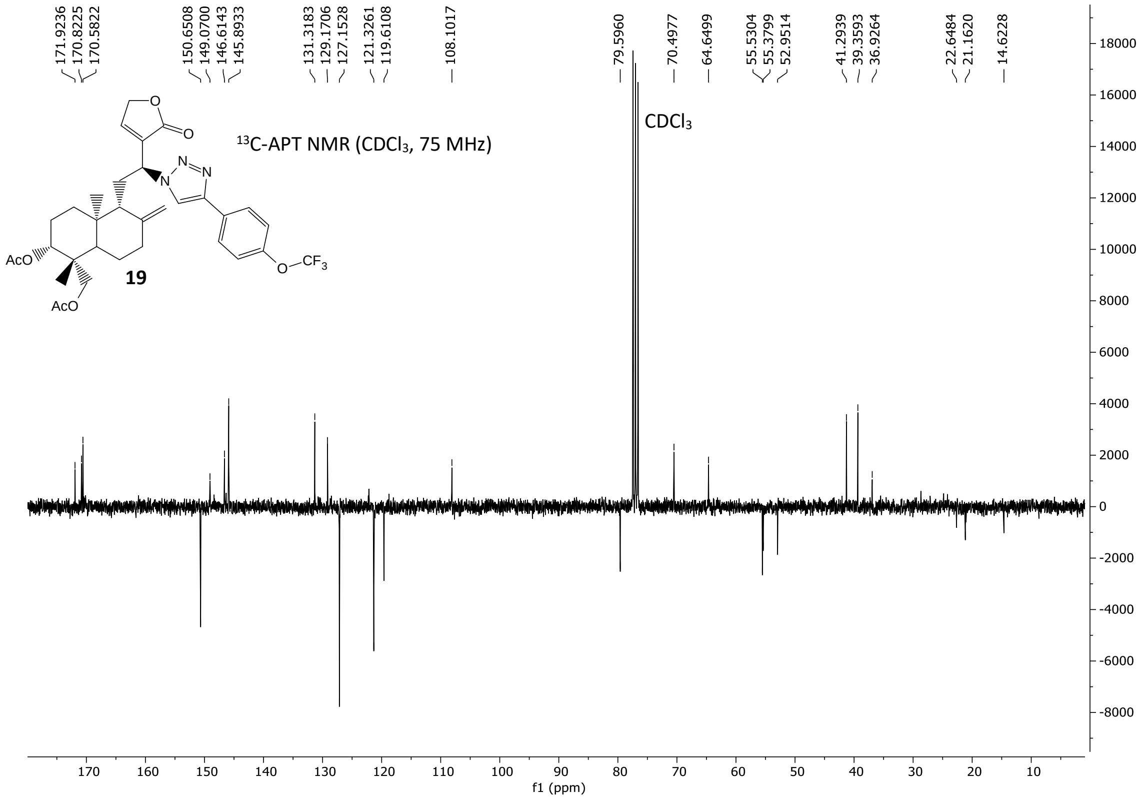

Figure S23: ^{13}C-APT NMR spectrum of compound **19** (75 MHz, $CDCl_3$). Quaternary (C) and methylene (CH_2) carbons are displayed as positive signals, while methine (CH) and methyl (CH_3) carbons are displayed as negative signals.

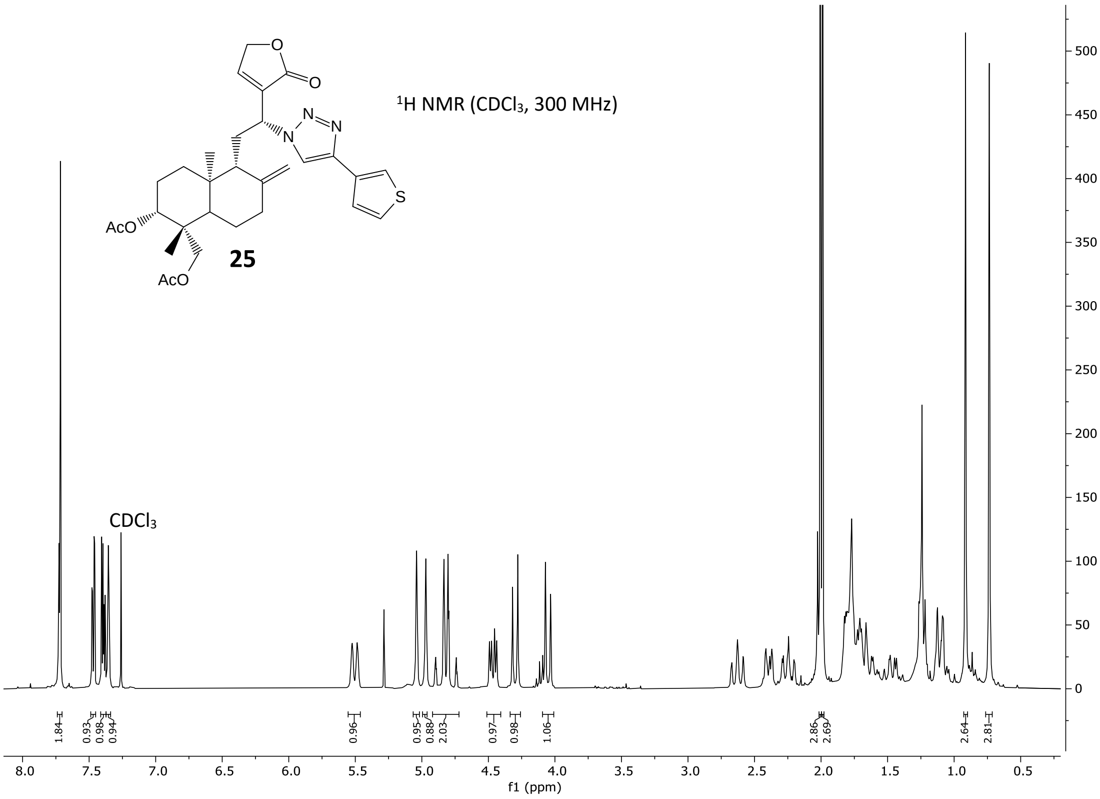

Figure S24: ^{1}H-NMR spectrum of compound **25** (300 MHz, $CDCl_3$)

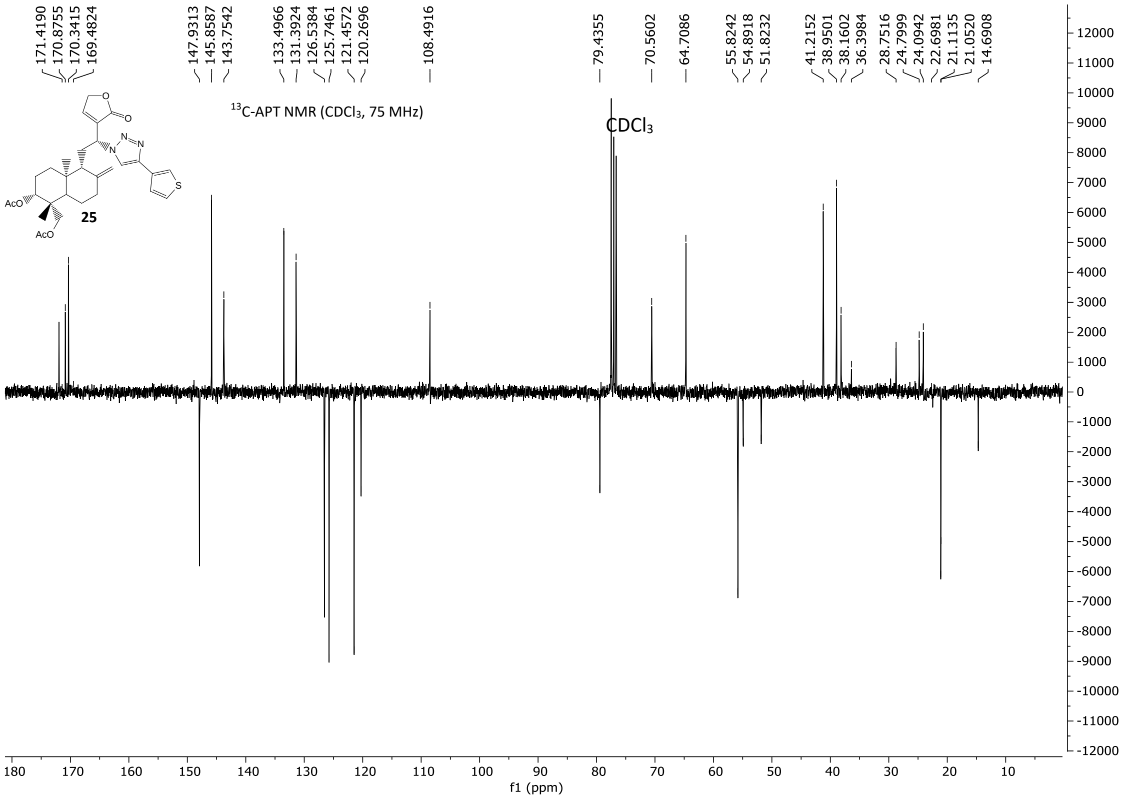

Figure S25: ^{13}C-APT NMR spectrum of compound **25** (75 MHz, $CDCl_3$). Quaternary (C) and methylene (CH_2) carbons are displayed as positive signals, while methine (CH) and methyl (CH_3) carbons are displayed as negative signals.

3. Uncropped blots

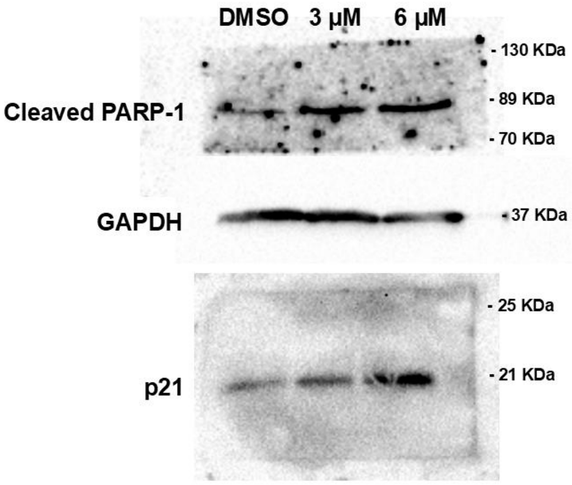

Figure S26: Uncropped blots showing cleaved PARP-1, p21 and GAPDH (used as a loading control) in PANC-1 cells treated with DMSO, 3 and 6 µM of compound **12**.

4. References

[1] A.E. Owen, H. Louis, E.U. Ejiofor, W. Emori, T.E. Gber, I. Benjamin, C.R. Cheng, M.M. Orosun, L. Ling, A.S. Adeyinka, Natural Andrographolide Isolated from Andrographis paniculata as Potent Epileptic Agent: Spectroscopy, Molecular Structure, and Molecular Docking Investigation, Chem. Africa. 6 (2023) 2445–2461. https://doi.org/10.1007/s42250-023-00657-9.

[2] E. Villedieu-Percheron, V. Ferreira, J.F. Campos, E. Destandau, C. Pichon, S. Berteina-Raboin, Quantitative Determination of Andrographolide and Related Compounds in Andrographis paniculata Extracts and Biological Evaluation of Their Anti-Inflammatory Activity, Foods. 8 (2019) 683–693. https://doi.org/10.3390/foods8120683.
